# Orexins contribute to restraint stress-induced cocaine relapse by endocannabinoid-mediated disinhibition of dopaminergic neurons

**DOI:** 10.1038/ncomms12199

**Published:** 2016-07-22

**Authors:** Li-Wei Tung, Guan-Ling Lu, Yen-Hsien Lee, Lung Yu, Hsin-Jung Lee, Emma Leishman, Heather Bradshaw, Ling-Ling Hwang, Ming-Shiu Hung, Ken Mackie, Andreas Zimmer, Lih-Chu Chiou

**Affiliations:** 1 Graduate Institute of Pharmacology, College of Medicine, National Taiwan University, Taipei 100, Taiwan; 2 Graduate Institute of Medical Sciences, College of Medicine, Taipei Medical University, No. 250 Wuxing Street, Taipei 11031, Taiwan; 3 Institute of Behavioral Medicine, College of Medicine, National Cheng Kung University, No.1, University Road, Tainan City 70101, Taiwan; 4 Department of Pharmacology, College of Medicine, National Taiwan University, Taipei 100, Taiwan; 5 Gill Center and the Department of Psychological and Brain Sciences, Indiana University, Bloomington, Indiana 47405, USA; 6 Department of Physiology, College of Medicine, Taipei Medical University, No. 250 Wuxing Street, Taipei 11031, Taiwan; 7 Institute of Biotechnology and Pharmaceutical Research, National Health Research Institutes, Zhunan, Miaoli County 35053, Taiwan; 8 Institute for Molecular Psychiatry, Medical Faculty, University of Bonn, 53127 Bonn, Germany; 9 Graduate Institute of Brain and Mind Sciences, College of Medicine, National Taiwan University, Taipei 100, Taiwan; 10 Research Center for Chinese Medicine & Acupuncture, China Medical University, Taichung 40447, Taiwan

## Abstract

Orexins are associated with drug relapse in rodents. Here, we show that acute restraint stress in mice activates lateral hypothalamic (LH) orexin neurons, increases levels of orexin A and 2-arachidonoylglycerol (2-AG) in the ventral tegmental area (VTA), and reinstates extinguished cocaine-conditioned place preference (CPP). This stress-induced reinstatement of cocaine CPP depends on type 1 orexin receptors (OX1Rs), type 1 cannabinoid receptors (CB1Rs) and diacylglycerol lipase (DAGL) in the VTA. In dopaminergic neurons of VTA slices, orexin A presynaptically inhibits GABAergic transmission. This effect is prevented by internal GDP-β-S or inhibiting OX1Rs, CB1Rs, phospholipase C or DAGL, and potentiated by inhibiting 2-AG degradation. These results suggest that restraint stress activates LH orexin neurons, releasing orexins into the VTA to activate postsynaptic OX1Rs of dopaminergic neurons and generate 2-AG through a G_q_-protein-phospholipase C-DAGL cascade. 2-AG retrogradely inhibits GABA release through presynaptic CB1Rs, leading to VTA dopaminergic disinhibition and reinstatement of cocaine CPP.

## Abstract (web summary)

Stress is a major cause of relapse to cocaine seeking behaviour. Tung *et al*. show that orexin mediates stress-induced reinstatement of cocaine seeking behaviour in mice by endocannabinoid-dependent disinhibition in the ventral tegmental area.

## Graphical abstract

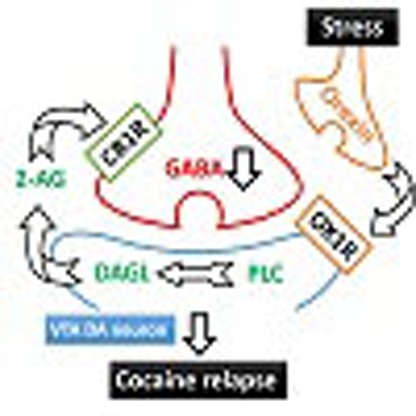

Even after extended periods of abstinence, drug relapse can be initiated by environmental cues, re-exposure to the drug or stress^1^,^2^. This severely limits the success of drug rehabilitation programs. Currently, there are few effective treatments to prevent drug relapse, which has an important socioeconomic impact.

The orexin system consists of orexin A and orexin B (ref. 3; also known as hypocretin 1 and hypocretin 2 (ref. 4)) and their G_q_ protein-coupled receptors (G_q_PCRs), orexin receptor type 1 (OX1R) and 2 (OX2R). Orexin neurons are restricted to the perifornical area (PeF), dorsomedial hypothalamus (DMH) and lateral hypothalamus (LH) in all mammals^5^, but project widely throughout the central nervous system^6^. In addition to mediating arousal, feeding and pain regulation^7^,^8^, orexins are also involved in reward^9^. The role of orexins in the reinstatement of drug seeking behaviours^10^ is especially noteworthy.

Orexin neurons in the LH send substantial projections to the ventral tegmental area (VTA)^11^, a brain region crucial for mediating natural and drug rewards^12^. Activation of LH orexin neurons is strongly associated with cue-reinstated drug and food seeking behaviours^13^. Additionally, intra-VTA or intracerebroventricular (i.c.v.) injection of orexin A induced cocaine or morphine seeking behaviours in extinguished rodents in an OX1R-dependent manner^6^. Moreover, the reinstatement of cocaine, alcohol, morphine or food seeking behaviours induced by cue, context, yohimbine or the rewarded drug was antagonized by an OX1R, but not OX2R, antagonist^6^. However it remains unclear whether orexins in the VTA are involved in stress-induced drug seeking^14^,^15^.

Several studies have investigated the cellular mechanisms in the VTA underlying orexin-induced reinstatement of reward seeking. Intra-VTA injection of orexin A increased extracellular dopamine levels in brain regions receiving dopaminergic VTA projections, the prefrontal cortex and nucleus accumbens^6^, suggesting orexin A increases dopaminergic activity in the VTA. Using VTA slices, Borgland *et al*.^16^ have demonstrated that orexin A increases VTA dopaminergic activity via long-term potentiation of glutamatergic transmission onto VTA dopaminergic neurons. Although this long-term plasticity in the VTA glutamatergic system may contribute to cocaine-induced sensitization^16^ or other drug rewards^17^, it may not be sufficient to stress induce drug relapse, since intra-VTA orexin A-induced reinstatement of cocaine seeking in rats is only partially glutamate-dependent^14^. In addition to glutamatergic signalling, GABAergic transmission is also an important modulator of VTA dopaminergic activity. Furthermore, application of orexin A to VTA slices, in addition to increasing the firing of dopaminergic neurons^18^, also directly increased the firing of GABAergic neurons. The latter is predicted to decrease VTA dopaminergic activity. Recently, Baimel *et al*.^19^ also reported that orexins may modulate a morphine-induced shift in the balance of excitatory and inhibitory synaptic inputs to VTA dopaminergic neurons. Therefore, it is likely that alternative mechanisms exist by which orexin A modulates GABAergic activity, and hence increases VTA dopaminergic activity.

Endocannabinoids have been implicated in the reinstatement of extinguished seeking behaviours for cocaine^20^, heroin^21^, ethanol^22^ and nicotine^23^ induced by cue, drug or forced swim stress since these reinstatements were blocked by a type 1 cannabinoid receptor (CB1R) antagonist. Endocannabinoids are synthesized on demand, especially 2-arachidonoylglycerol (2-AG), an endocannabinoid that can be generated when certain G_q_PCRs are activated, including OX1Rs (ref. 24), and produces retrograde inhibition of neurotransmitter release^25^.

OX1Rs are abundantly distributed in VTA dopaminergic neurons^18^, and the 2-AG synthesizing enzyme, diacylglycerol lipase (DAGL) is located postsynaptically in these neurons, apposed to the CB1R-expressing GABAergic terminals^26^. Therefore, we hypothesize that activation of postsynaptic OX1Rs in VTA dopaminergic neurons stimulates the G_q_PCR-mediated phospholipase C (PLC)-DAGL enzymatic cascade and generates 2-AG that produces retrograde inhibition of GABA release by activating CB1Rs located on GABA terminals impinging on VTA dopaminergic neurons. Here, we validate this hypothesis through electrophysiological recordings in VTA slices. We further establish that this OX1R-PLC-DAGL-2-AG cascade-mediated disinhibition of VTA dopaminergic neurons contributes to acute restraint stress-induced reinstatement of extinguished cocaine conditioned place preference (CPP) in mice, an animal model that mimics stress-induced cocaine relapse.

## Results

### Orexin A depressed IPSCs of VTA dopamine neurons via OX1Rs

**Figure 1:**
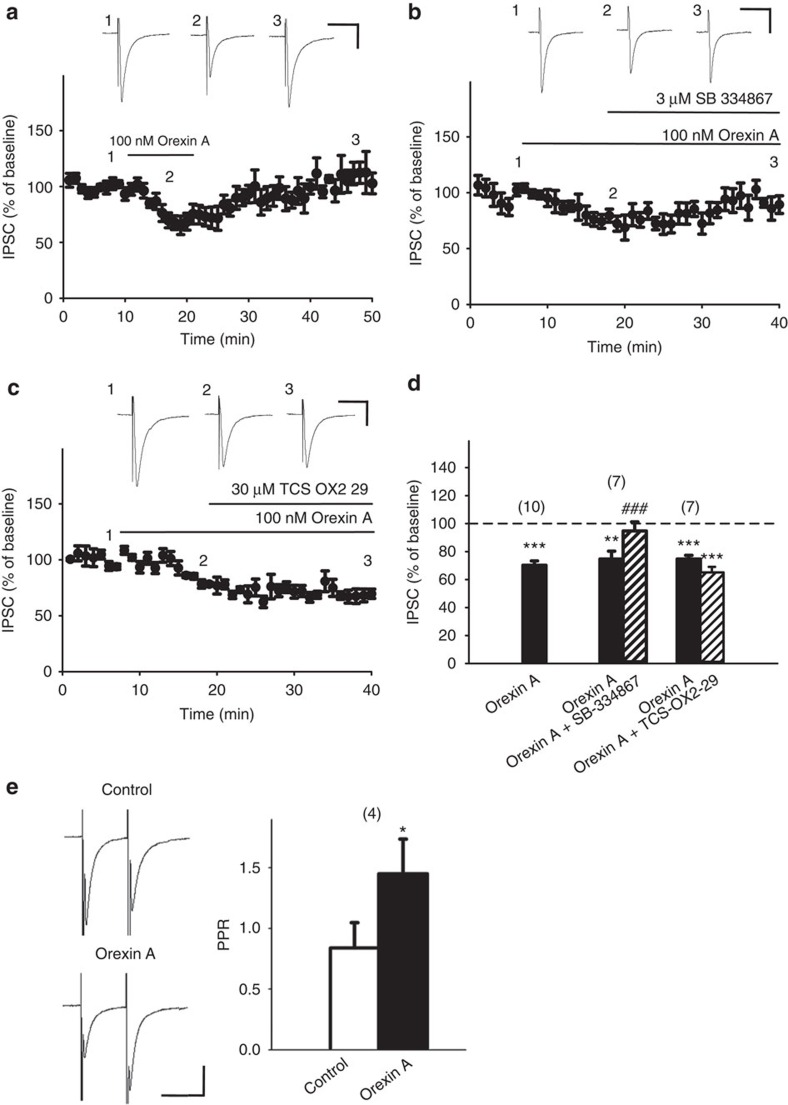
Orexin A depressed IPSCs in VTA slices through OX1Rs, but not OX2Rs, via a presynaptic mechanism. (**a**–**c**) Time courses of the effects of orexin A on IPSC amplitude in representative slices (**a**) alone, or in combination (**b**) with SB-334867, an OX1R antagonist, or (**c**) with TCS-OX2-29, an OX2R antagonist. Inward IPSCs evoked at 0.05 Hz were recorded at −70 mV with a KCl-based internal solution in the presence of 2 mM kynurenic acid, and every three IPSCs were averaged. Shown are representative traces taken at the indicated time points. (**d**) Summarized effects of orexin A (100 nM) on IPSC amplitude without (black bars) or with 3 μM SB-334867 (*n*=7, *P*<0.001 ; *t*(6)=6.818) or 30 μM TCS-OX2-29 (*n*=7, *P*=0.0814; *t*(6)=2.091). Ten IPSCs at 10–15 min after drug application, when the response had stabilized, were averaged. The averaged amplitude of 10 IPSCs before drug treatment in each neuron was taken as 100% baseline. Data are presented as mean±s.e.m. The number of recorded neurons is denoted above each bar. Grouped bars represent the data from the same group with different treatments conducted sequentially. **P<0.01, ***P<0.001 versus 100% (one sample *t*-test); ^###^*P*<0.001 versus orexin A alone: *n*=10 (paired *t*-test). (**e**) The effect of orexin A (100 nM) on the PPR of IPSCs. Paired IPSCs were evoked by 50 ms-separated paired pulses every 20 s. Left panel: a representative paired IPSCs recorded in a neuron before and 10 min after orexin A treatment. Scale bars, 50 ms and 200 pA. Right panel: average PPR of IPSCs in four neurons recorded before (control) and after orexin A treatment. (*n*=4, *P*=0.026; *t*(3)=4.089). *P<0.05 versus control (paired *t*-test). Data are expressed as mean±s.e.m.

Inhibitory GABAergic postsynaptic currents (IPSCs; Fig. 1) evoked by rostral stimulation in rat VTA dopaminergic neurons were recorded in the presence of 2 mM kynurenic acid, an ionotropic glutamate receptor blocker^27^. Dopaminergic neurons were identified by the presence of hyperpolarization-activated current, low spontaneous single-pacemaker firings^28^ and tyrosine hydroxylase immunoreactivity (Supplementary Fig. 1).

Orexin A (100 nM) significantly depressed IPSCs (Fig. 1) in 30 out of 32 (94%) recorded VTA dopaminergic neurons (a depression of >10% was taken as a positive response). In many (16/32, 50%) of these neurons, orexin A also induced a significant inward current (55.38±10.17 pA). The IPSC depressant effect of orexin A was reversible (Fig. 1a), and persisted during the 30 min-application period (Supplementary Fig. 2). IPSC inhibition by orexin A was reversed by SB-334867 (3 μM), an OX1R antagonist (Fig. 1b,d), but not by TCS-OX2-29 (30 μM), a selective OX2R antagonist (Fig. 1c,d). Pretreatment with SB-334867 had no effect on IPSCs *per se*, but prevented orexin A (100 nM)-induced IPSC depression. IPSCs after treatment with SB-334867 alone or in combination with orexin A were not significantly different (114.1±7.0 versus 127.1±11.3% of baseline, *n*=5, paired *t*-test, *P*=0.362, *t*(4)=1.027). These results suggest that orexin A depressed IPSCs in VTA dopaminergic neurons through OX1Rs, and not through OX2Rs.

### Orexin A inhibited GABAergic transmission presynaptically

**Figure 2:**
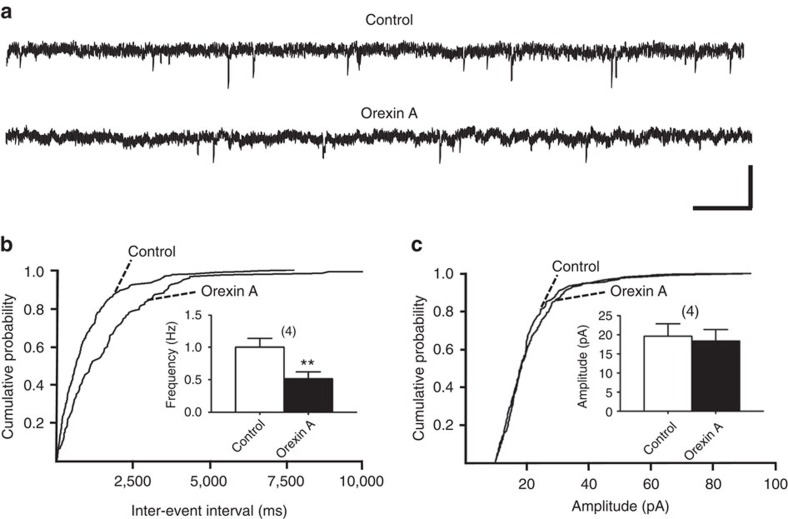
Orexin A decreased the frequency, but not the amplitude, of mIPSCs. (**a**) Representative traces of mIPSCs recorded in the presence of 2 mM kynurenic acid and 1 μM tetrodotoxin in a neuron before (upper panel) and 10 min after (lower panel) treatment with 100 nM orexin A. Scale bars, 0.5 s and 50 pA. (**b**,**c**) Cumulative probability of the interval or amplitude of mIPSCs, sampled in a 5-min period, before and 10 min after treatment with 100 nM orexin A (black bars). Insets: (**b**) the average frequency (*n*=4, *P*=0.0028 ; *t*(3)=9.127) and (**c**) amplitude (*n*=4, *P*=0.1142 ; *t*(3)=2.209) of mIPSCs before and 10 min after treatment with orexin A in the same neurons. **P<0.01 versus control (paired *t*-test). Data are expressed as mean±s.e.m.

Next, we examined the effect of orexin A on the paired-pulse ratio (PPR) of paired IPSCs, a phenomenon primarily interrogating presynaptic release machinery^29^. Orexin A (100 nM) depressed both the first and second IPSCs elicited by a 50 ms-separated paired pulse, while significantly increasing the PPR of paired IPSCs (Fig. 1e). Additionally, orexin A (100 nM) significantly reduced the frequency (Fig. 2a,b), but not the amplitude (Fig. 2a,c), of miniature IPSCs (mIPSCs), leading to a rightward shift in the cumulative distribution of mIPSC intervals (*P*<0.01, Kolmogorov–Smirnov test; Fig. 2b), but not amplitude (Fig. 2c). These results suggest that orexin A inhibits GABAergic transmission in VTA dopaminergic neurons by decreasing GABA release.

### Orexin A inhibited GABA release through CB1Rs

Orexin A is excitatory to neurons^7^, suggesting it inhibits GABA release indirectly. We have previously found that orexin A reduced GABA release through endocannabinoid retrograde signalling in the periaqueductal grey^24^ and here we examined if this mechanism also exists in the VTA.

**Figure 3:**
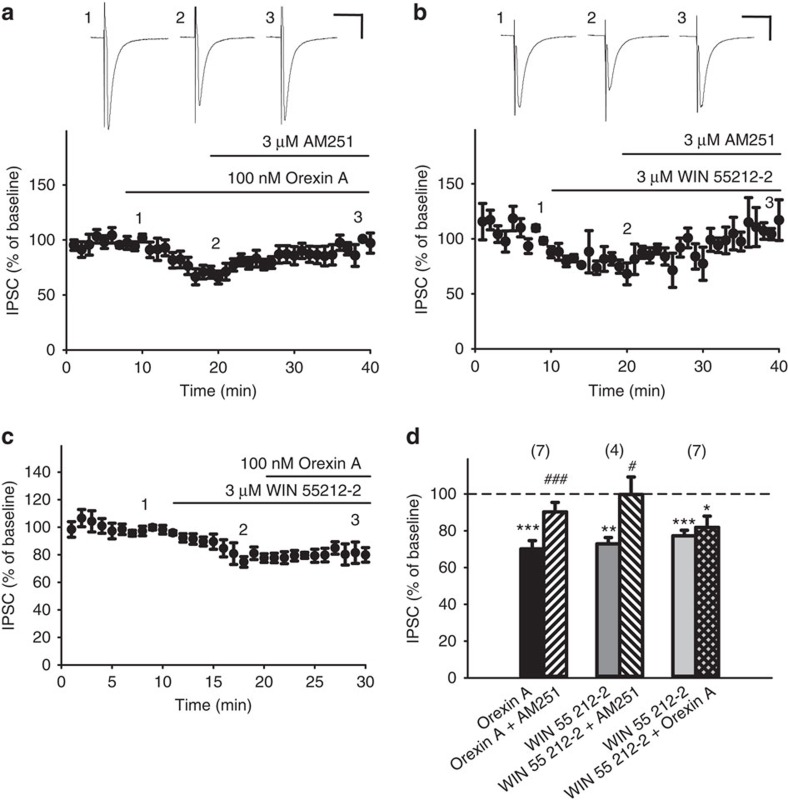
Orexin A-induced IPSC depression was reversed by a CB1R antagonist, and was mimicked and occluded by a CB1R agonist. (**a**–**c**) The time course of the effect of (**a**) orexin A (100 nM) or (**b**) WIN 55,212-2 (3 μM) on IPSC amplitude before and after further treatment with AM 251 (3 μM), or with (**c**) orexin A. (**d**) The effect of orexin A (black bar) or WIN 55,212-2 (grey bars) alone or in combination with AM 251 (slashed bars; *n*=7, *P*<0.001 ; *t*(6)=−10.433 versus *n*=4, *P*=0.0481; *t*(3)=−3.232) or WIN 55212-2+orexin A (crossed bars; *n*=7, *P*=0.569 ; *t*(6)=−0.602) on IPSC amplitude. Scale bars, 50 ms and 200 pA. *P<0.05, **P<0.01, ***P<0.001 versus 100% (One sample *t*-test); ^#^*P*<0.05, ^###^*P*<0.001 versus Orexin A alone (paired *t*-test). Data are expressed as mean±s.e.m.

Orexin A (100 nM)-induced IPSC depression was reversed by the CB1R antagonist, AM 251 (3 μM; Fig. 3a,d). WIN 55212-2 (3 μM), a synthetic cannabinoid, also depressed IPSCs in a manner reversed by AM 251 (3 μM) (Fig. 3b,d). Pretreatment of slices with AM 251 had no effect on IPSCs *per se* but prevented orexin A (100 nM)-induced IPSC depression. The amplitude of IPSCs after AM 251 and AM 251+orexin A treatment were not significantly different (97.7±4.6 versus 125.9±15.0% of baseline, *n*=5, paired *t*-test, *P*=0.09, *t*(4)=−2.175). Importantly, pretreatment with WIN 55212-2 (3 μM) occluded the effect of subsequent orexin A (100 nM) treatment (Fig. 3c,d). These results suggest that orexin A, through OX1R, reduces GABA release via endocannabinoids acting at presynaptic CB1Rs on GABAergic terminals that innervate VTA dopaminergic neurons^30^.

### Orexin A depressed IPSCs via a GqPCR-PLC-DAGL-2AG cascade

**Figure 4:**
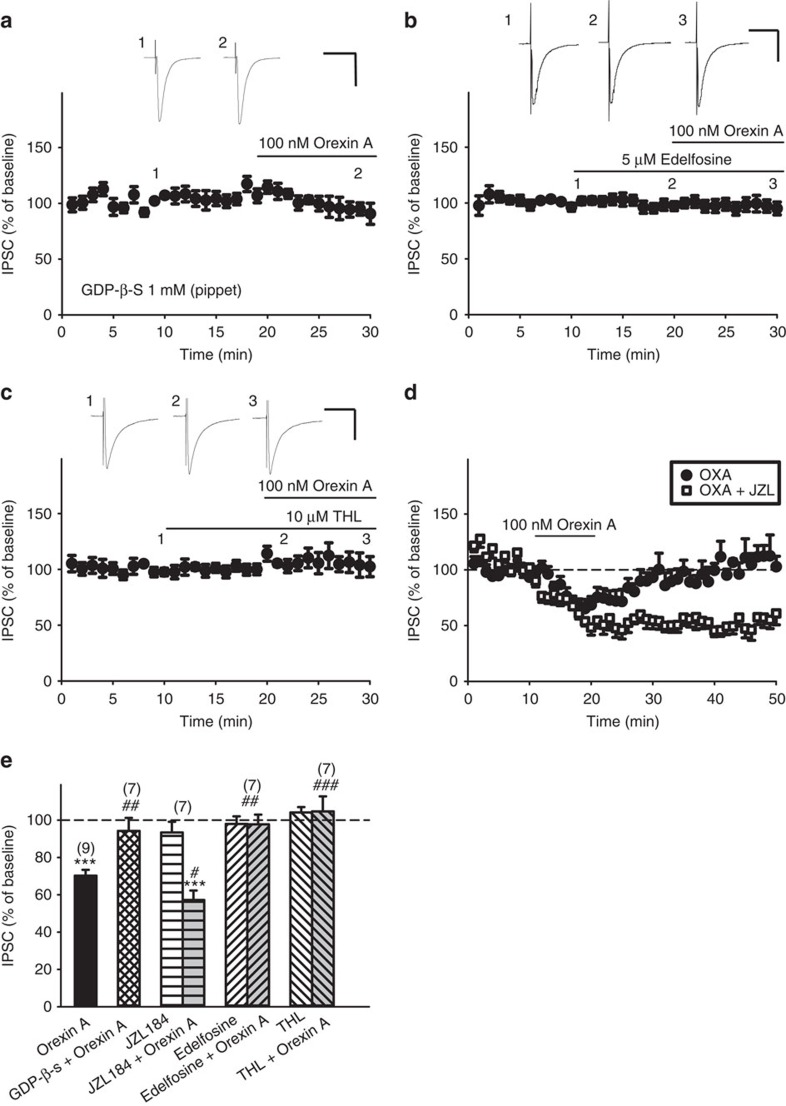
Orexin A-induced IPSC depression was prevented by inhibitors of G-proteins, PLCβ or DAGL, and was enhanced by a MAGL inhibitor. (**a**–**c**) The time course of the effect of orexin A on IPSC amplitude in slices pretreated with (**a**) GDP-β-S, a non-selective G protein inhibitor that was applied intracellularly through the patch pipette, (**b**) edelfosine (a PLCβ inhibitor) or (**c**) THL (a DAGL inhibitor). Scale bars, 50 ms, and (**a**) 400 pA, (**b**) 100 pA and (**c**) 200 pA. (**d**) The time course of the effect of orexin A on IPSC amplitudes alone (filled circles) or in the presence of 1 μM JZL184 (open squares), a selective inhibitor of MAGL, which is the major catabolic enzyme of 2-AG. (**e**) Summarized effects of orexin A on IPSC amplitude in the absence (*n*=9, black bar) or presence of 1 mM GDP-β-S (hatched bar; *n*=7, *P*=0.005 ; *t*(14)=−3.378), 5 μM edelfosine (right slashed grey bar; *n*=7, *P*=0.038; *t*(14)=2.286), 10 μM THL (left slashed grey bar; *n*=7, *P*<0.001 ; *t*(14)=−4.755) or 1 μM JZL184 (horizontal-lined bar; *n*=7, *P*<0.001 ; *t*(14)=−4.382), and the effect of the inhibitor alone (left or right slashed bar). ^***^P<0.001 (one sample *t*-test); ^#^*P*<0.05, ^##^*P*<0.01, ^###^*P*<0.001 versus the group treated with orexin A alone (Student's *t*-test). Data are expressed as mean±s.e.m.

Orexin A (100 nM)-induced IPSC depression was prevented by intracellular dialysis with GDP-β-S (1 mM; Fig. 4a,e), a nonhydrolyzable GDP analogue that decreases G protein activity. The effect of orexin A was also prevented by pretreatment with 5 μM edelfosine, a PLC inhibitor^31^ (Fig. 4b,e), or 3 μM (−)-tetrahydrolipstatin (THL), an inhibitor of DAGL (ref. 32), a 2-AG synthesizing enzyme (Fig. 4c,e). These enzyme inhibitors *per se* had no effect on IPSCs (Fig. 4b,c,e). In addition, JZL184 (1 μM), a selective inhibitor of monoaclyglycerol lipase (MAGL)^33^ is a major degrading enzyme of 2-AG (ref. 34), significantly potentiated and prolonged the effect of orexin A (Fig. 4d,e). These results suggest that orexin A inhibits GABAergic transmission, that is, induces disinhibition, indirectly via 2-AG, an endocannabinoid that is synthesized via a G_q_ protein-coupled PLC-DAGL enzymatic cascade and is degraded by MAGL.

### Orexin A did not depress EPSCs in VTA dopaminergic neurons

CB1Rs are also located on some glutamatergic terminals impinging onto VTA dopaminergic neurons^26^. Therefore, we also examined the effect of orexin A on excitatory postsynaptic currents (EPSCs) in VTA dopaminergic neurons. Orexin A did not depress and even tended to enhance EPSCs (Supplementary Fig. 3), which were recorded in the presence of 10 μM bicuculline. This is consistent with the findings of Borgland *et al*.^16^ that orexin A did not acutely affect AMPAR-mediated EPSCs.

### Orexin A increased VTA neuronal firing indirectly via CB1Rs

**Figure 5:**
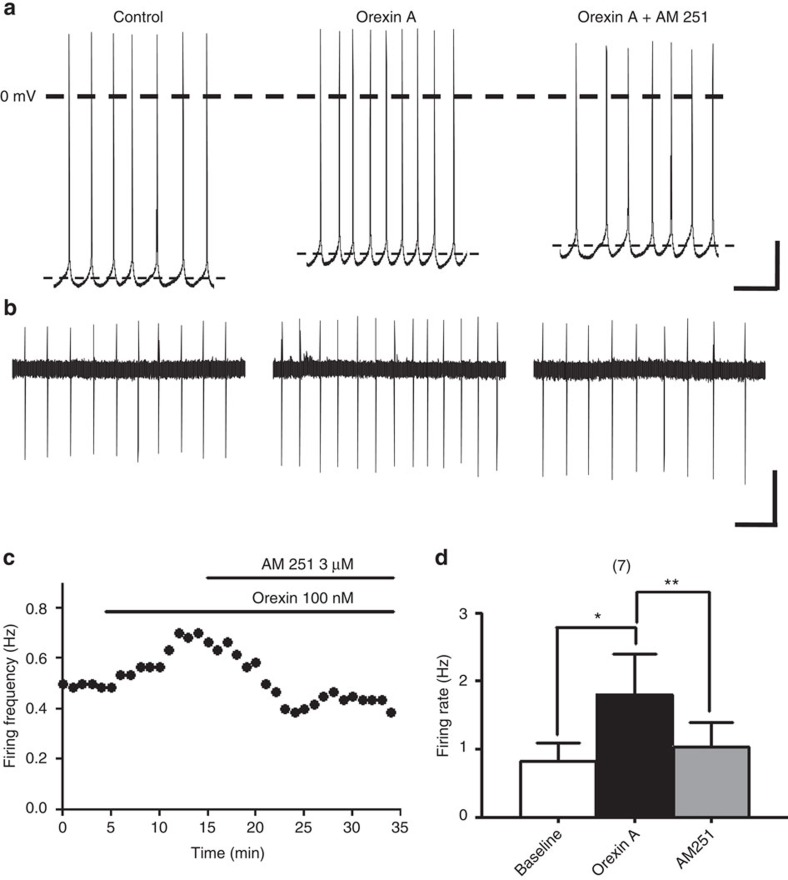
Orexin A increased the firing rate of VTA dopaminergic neurons through CB1Rs. (**a**,**b**) The firing activity in a representative neuron recorded in the (**a**) whole cell or (**b**) cell-attached recording mode before and 10 min after treatment with 100 nM orexin A, and 10 min after further treatment with 3 μM AM 251. Scale bars, (**a**) 500 ms and 20 mV; (**b**) 5 s and 20 pA. (**c**) The time course of the effect of orexin A before and after further treatment with AM 251 on the firing rate of a representative neuron recorded by cell-attached mode. (**d**) Effects of orexin A alone or in combination with AM 251 on the average firing rate of VTA dopaminergic neurons. Neuronal firing activity was assessed in the whole cell and cell-attached recording mode. Note that 100 nM orexin A (black bar) significantly increased the average firing rate of VTA dopaminergic neurons, compared with baseline firing rate (open bar) and this effect was reversed by 3 μM AM 251 (gray). *n*=7, *P<0.05, **P<0.01 (repeat measures one-way ANOVA with Dunns *post hoc* test). Data are expressed as mean±s.e.m.

To substantiate the functional role of 2-AG-mediated disinhibition in regulating the neuronal activity of VTA dopaminergic neurons, we examined the effect of orexin A on the firing activity of dopaminergic neurons in VTA slices without any receptor blockers in the whole-cell current-clamp recording mode. Orexin A (100 nM) increased the firing rate of dopaminergic neurons (Fig. 5a). Orexin A also induced membrane depolarization in 4 out of 5 recorded neurons, changing the membrane potential from −57.49±3.8 mV to −47.92±1.9 mV, as reported previously^18^. Importantly, orexin A-induced elevation of neuronal activity, but not membrane depolarization, was robustly reversed by AM 251 (3 μM) (Fig. 5a). Orexin A (100 nM)-induced membrane depolarization in all recorded neurons (9.56±4.3 mV) was not significantly decreased (paired *t*-test, *P*=0.10, *t*(4)=2.178) after further treatment with AM 251 (16.53±7.4 mV).

In the whole-cell recording mode, the firing frequency of VTA dopaminergic neurons may change due to a dialysis of intracellular components after long-term recording^35^. We therefore quantified neuronal firing frequency in the cell-attached recording mode. The firing rate in all seven recorded neurons was significantly increased by orexin A (100 nM), and reduced to basal levels by further application of AM 251 (Fig. 5b–d).

These results suggest that orexin A increases the firing rate of VTA dopaminergic neurons indirectly through a CB1R-dependent mechanism, likely via the 2-AG-mediated inhibitory effect on GABAergic transmission. Although orexin A also induced postsynaptic depolarization that may increase neuronal firing rate, this depolarization effect is CB1R-independent.

### The OX1R-2-AG-CB1R cascade in stress-induced cocaine relapse

We next determined if endogenous orexins are released under certain circumstances to induce disinhibition of VTA dopaminergic neurons via the OX1R-PLC-DAGL-2-AG cascade that was revealed in VTA slices. Our focus was on stress, which can induce reinstatement of extinguished drug seeking behaviours. Hypothalamic orexin neurons can be activated by diverse stressors, such as restraint-induced stress^36^,^37^. We therefore examined if OX1R- and CB1R-mediated disinhibition of VTA dopaminergic activity contributes to stress-induced cocaine relapse, using a 30-min-restraint stress to reinstate extinguished cocaine CPP in mice (Methods section). First, we confirmed that, in mouse VTA slices, orexin A (100 nM) also depressed IPSCs in dopaminergic neurons in a manner reversed by AM 251 (3 μM; Supplementary Fig. 4), as observed in rat VTA slices.

### Restraint stress reinstated extinguished cocaine CPP in mice

**Figure 6:**
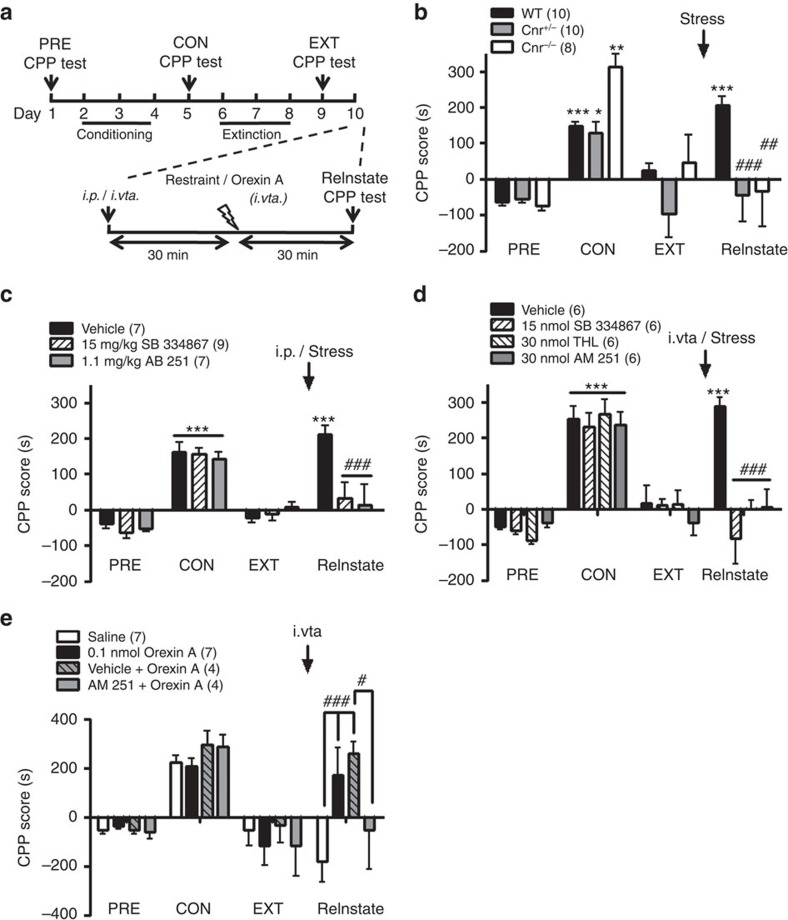
Restraint stress or intra-VTA microinjection of orexin A reinstated extinguished cocaine-CPP in WT, but not in *Cnr1 KO* mice or in mice pretreated with an OX1R or CB1R antagonist. (**a**) The protocol for inducing, extinguishing, and reinstating cocaine-CPP. Four stages were analysed: preconditioning^1^, conditioning^35^, extinction and reinstatement. Mice received daily pairing of cocaine (i.p. 20 mg kg^−1^) on days 2–4 to develop cocaine CPP, followed by a 3-day forced extinction training by daily paring of saline with the cocaine-paired chamber on days 6–8. (**b**–**e**) On day 10, (**b**–**d**) a 30-min restraint stress or (**e**) bilateral intra-VTA microinjection (i.vta.) of 0.1 nmol orexin A for 30 min was applied before the CPP test to reinstate extinguished cocaine CPP. The CPP test was conducted before (day 1, PRE) and after (day 5, CON) cocaine-conditioning, after extinction (day 9, extinction) and after restraint stress/i.vta. orexin A (day 10, reinstatement). Antagonists were given 30 min before restraint stress by either i.p. injection or bilateral i.vta. microinjection. The degree of cocaine seeking was determined by the CPP score as described in Methods section. (**b**–**d**) CPP scores at various stages (**b**) in WT (*n*=10, black bars), *Cnr1*^*+/*−^ (*n*=10, grey bars) or *Cnr1*^−*/*−^ (*n*=8, open bars) mice, or in mice (**c**) i.p. or (**d**) i.vta. pretreated with vehicle (i.p.: *n*=7, i.vta: *n*=6, black bars), SB-334867 (i.p.: *n*=9, i.vta: *n*=6, left slashed bars), AM 251 (i.p.: *n*=7, i.vta: *n*=6, grey bars) or THL (*n*=6, right slashed bars) before receiving restraint stress. (**e**) CPP scores at various stages in mice, after extinction, i.vta. microinjection of orexin A (*n*=7, black bars), saline (*n*=7, open bars), orexin A+AM 251 (*n*=4, grey bars) or orexin A+vehicle (*n*=4, right slashed grey bars). *P<0.05, **P<0.01, ***P<0.01 versus the PRE stage ; ^#^*P*<0.05, ^##^*P*<0.01, ^###^*P*<0.001 versus (**b**–**d**) the WT, or the vehicle or (**e**) the saline group (two-way ANOVA with Bonferroni *post hoc* test). Data are expressed as mean±s.e.m.

Figure 6a shows the protocol used for establishing the cocaine CPP model that included four stages: pre-conditioning (PRE), cocaine conditioning (CON), extinction (EXT) and reinstatement (ReInstate). In wild-type (WT) mice (black bars in Fig. 6b), cocaine CPP was successfully induced by pairing cocaine (20 mg kg^−1^) for 3 days, and then extinguished by pairing saline for 3 days as described in Methods section. After the extinction, a 30-min restraint stress reinstated cocaine CPP (Fig. 6b), that is, induced cocaine relapse. The CPP score, which had been elevated during the CON stage, was reduced back to the pre-conditioning level in the extinction stage and was elevated again after stress to a similar level as at the CON stage.

### Stress did not induce cocaine relapse in *Cnr1*-deficient mice

In all *Cnr1*-deficient mice, either hetero- or homo-zygous, cocaine-conditioning induced similar CPP scores as in WT mice (grey versus black bars; open versus black bars; open versus grey bars, CON, Fig. 6b, Bonferroni *post hoc* analysis, *P*>0.05), suggesting cocaine CPP is also successfully induced in *Cnr1*-deficient mice, as reported previously^38^,^39^. Likewise, all *Cnr1*-deficient mice displayed negligible cocaine CPP following the extinction training, similar to WT mice (EXT, Fig. 6b). However, acute restraint stress failed to reinstate cocaine CPP in either group of mutant mice (ReInstate, Fig. 6b). A two-way analysis of variance (ANOVA) with repeated measures over the stages shows a main effect of stage (F_(3,75)=_21.02, *P*<0.001) and a significant interaction between genotype and stage (F_(6,75)_=4.698, *P*<0.001). Bonferroni *post hoc* analysis shows a significant difference between genotypes at the reinstatement stage (*P*<0.01), but not at the PRE, CON or extinction stage.

### Systemic OX1R and CB1R blockade prevented cocaine relapse

Next, we used pharmacological approaches to examine the role of OX1Rs and CB1Rs in restraint stress-induced reinstatement of cocaine CPP. Mice were randomly divided into three groups, receiving i.p. injection of vehicle, SB-334867 (15 mg kg^−1^) or AM 251 (1.1 mg kg^−1^) 30 min before restraint stress. Before drug treatments, all three groups of mice received similar cocaine-conditioning and extinction training and showed comparable CPP scores at PRE, CON and EXT stages (Fig. 6c). Restraint stress reinstated cocaine CPP to the level before extinction in vehicle-pretreated extinguished mice, but had no effect in SB-334867- or AM 251-pretreated extinguished mice (Fig. 6c, two-way ANOVA for stage (F_(3,60)=_39.68, *P*<0.001), and interaction between treatment and stage (F_(6,60)_=4.383, *P*<0.001)).

### VTA OX1Rs, CB1Rs and DAGL in stress-induced cocaine relapse

To verify the site of action of the antagonist, we microinjected SB-334867 (15 nmol), AM 251 (30 nmol) or THL (30 nmol), a DAGL inhibitor, bilaterally into the VTA (i.vta.) of mice 30 min before restraint stress. All three i.vta. pretreatments prevented stress-induced reinstatement (*P*<0.001 versus vehicle) (Fig. 6d, two-way ANOVA for stage (F_(3,60)=_67.19, *P*<0.001) and interaction between treatment and stage (F_(9,60)_=6.148, *P*<0.001)).

### Orexin A (i.vta.) induced cocaine relapse via CB1Rs

Next, we examined whether extinguished cocaine CPP can be reinstated by microinjection of exogenous orexin A into the VTA, through the CB1R signalling. Intra-VTA microinjection of orexin A (0.1 nmol), but not saline, in extinguished mice significantly reinstated cocaine CPP (Fig. 6e). This effect of i.vta. orexin A was antagonized by i.vta. co-treatment with AM 251 (30 nmol), but not with by vehicle (Fig. 6e, two-way ANOVA for treatment (F_(3,54)=_21.01, *P*<0.001); for the interaction between treatment and stage (F_(9,54)_=2.77, *P*<0.01)).

### Acute restraint stress activated LH orexin neurons

**Figure 7:**
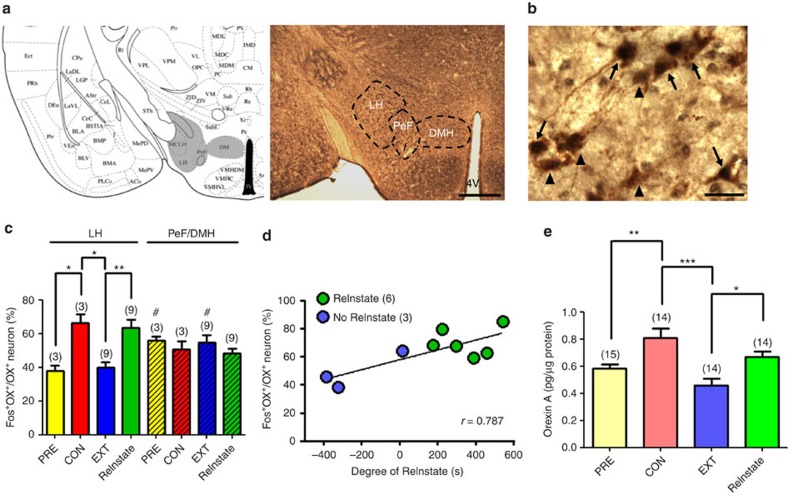
Restraint stress increased the number of c-fos-expressing orexin neurons in the LH and orexin A levels in the VTA. (**a**,**b**) A representative hypothalamic section taken at the level of (**a**) the LH and PeF/DMH or (**b**) the LH (reproduced from ref. 52) from a pre-conditioned mouse. Scale bars: (**a**) 300 μm, (**b**) 20 μm. Orexin A (OX^+^) was labelled by DAB (ref. 18) and c-fos (Fos^+^) was labelled by DAB with nickel (black in nucleus). Arrows indicate neurons doubly immunoreactive for orexin A and c-fos (Fos^+^OX^+^). Arrowheads indicate orexin neurons without c-fos staining. 4 V: the fourth ventricle. (**c**) The number of Fos^+^OX^+^ neurons as a percentage of total OX^+^ neurons, which reflects the percentage of activated orexin neurons, in the LH or PeF/DMH at each stage (PRE: *n*=3; CON: *n*=3; extinction: *n*=9; reinstatement: *n*=9, *P<0.05; **P<0.01, one-way ANOVA with Newman–Keuls *post hoc* test; ^#^*P*<0.05, paired *t*-test). (**d**) The association between the number of activated LH orexin neurons and the degree of reinstatement, expressed by the CPP score difference between reinstatement and extinction stages. Note that there is a positive linear correlation between the number of activated orexin neurons and the degree of reinstatement of cocaine CPP in mice whether (green dots) or not (blue dots) restraint stress reinstated cocaine CPP. *n*=9, *r*=0.787, *P*<0.05. (**e**) The average level of orexin A in the VTA homogenate at each stage of cocaine CPP. (PRE: *n*=15; CON: *n*=14; extinction: *n*=14; reinstatement: *n*=14, *P<0.05; **P<0.01; ***P<0.001, one-way ANOVA with Newman–Keuls *post hoc* test). Data are expressed as mean±s.e.m.

Harris *et al*.^9^,^40^ have reported that rats in a drug-associated environment display more c-fos-containing orexin neurons retrogradely labelled from the VTA in the LH, but not PeF or DMH (ref. 40), than control rats. To further validate that restraint stress can activate hypothalamic orexin neurons, we measured the percentage of activated orexin neurons in the LH and PeF/DMH (Fig. 7a) of mice at each stage of the cocaine CPP model (Fig. 6a). Activated orexin neurons (arrows in Fig. 7b) were identified by the presence of double immunoreactivity to c-fos (localized in the nucleus, black) and orexin A (localized in the cytosol, brown) in hypothalamic slice sections, whereas non-activated orexin neurons only showed brown cytosol (arrowheads in Fig. 7b).

Mice were randomly divided into four groups and killed at one of four stages of the cocaine CPP model. The percentage of c-fos-expressing orexin neurons in the LH of mice at the CON stage was significantly higher than at the PRE stage (Fig. 7c). At the EXT stage, it was decreased to the level of the PRE stage, and then increased at the ReInstate stage to the level of the CON stage (Fig. 7c, one-way ANOVA with Newman–Keuls *post hoc* correction, F_(3,23)=_9.026, *P*=0.0006). An additional non-stressed control group, which received the same CPP, extinction and reinstatement procedures as the restraint group, but were not subjected to the 30-min-restraint stress, also showed significantly fewer activated orexin neurons than the restraint group in the reinstatement stage (42.5±2.0 versus 63.4±14.7% of orexin neuron activated, Students *t*-test, *P*<0.05, *t*(10)=2.377). Importantly, the degree of reinstatement in each mouse, calculated by the CPP score difference between ReInstate and EXT stages, was positively correlated with the percentage of activated LH orexin neurons (Pearson correlation, *r*=0.787, *P*<0.05, Fig. 7d). In contrast to the LH, orexin neurons in the PeF/DMH displayed no significant activation in mice at any stage in this cocaine CPP model (F_(3,23)=_0.8324, *P*=0.4918, Fig. 7c).

### Acute restraint stress elevated VTA orexin A and 2-AG levels

Figure 7e shows the mean orexin A level in VTA homogenates prepared from mice at different stages of the cocaine CPP model. One-way ANOVA revealed a significant difference among groups (F_(3,53)=_8.495, *P*=0.0001). *Post hoc* Newman–Keuls analysis shows that orexin A levels in mice at the CON stage were significantly increased, as compared with the PRE stage, and at the EXT stage were significantly decreased, back to PRE stage levels. Furthermore, at the ReInstate stage, VTA orexin A levels were significantly elevated, compared with the EXT stage. The non-stressed control group also had significantly lower VTA orexin A levels compared with the restraint group in the ReInstate stage (0.54±0.10 versus 0.67±0.15 pg μg^−1^ protein, Students's *t*-test, *P*<0.05, *t*(23)=2.538).

We further measured various endocannabinoids in the VTA of mice at the reinstatement stage by high pressure liquid chromatography coupled with tandem mass spectrometry (HPLC/MS/MS; Supplementary Table 1). The level of 2-AG in the VTA of restrained mice was significantly higher than that in the non-restrained group (547.65±42.73 versus 422.95±59.93 pmol g^−1^-tissue, Mann–Whitney *U* test, *P*<0.05, *t*(14)=1.694).

## Discussion

**Figure 8:**
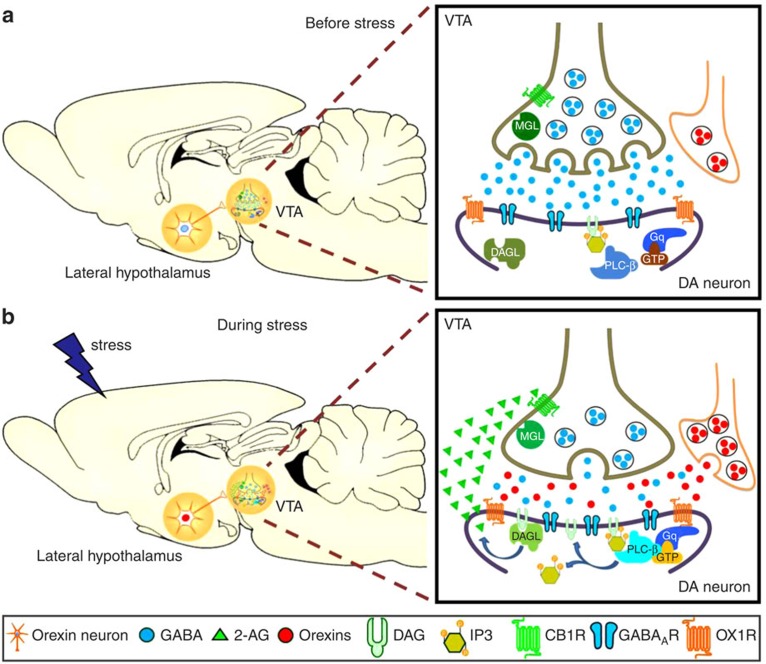
A schematic illustration of how stress-released orexins induce an endocannabinoid-mediated relapse for cocaine-seeking. This scheme was illustrated by using the Biology Bundle of Motifolio PPT Drawing Toolkits (Motifolio Inc, MD) to describe what may occur in the LH and the VTA (**a**) before stress and (**b**) during stress. Right boxes are enlarged portions of synaptic events occurring in a GABAergic synapse onto a dopaminergic neuron in the VTA. During stress, LH orexin neurons are activated and release orexins. The released orexins then activate postsynaptic OX1 receptors on dopaminergic neurons in the VTA. Activation of the OX1 receptor, a G_q_-protein coupled receptor, leads to PLC activation, generating DAG that is converted into 2-AG, an endocannabinoid, by DAGL. 2-AG travels retrogradely across the synapse to inhibit GABA release by activating presynaptic CB1 receptors on the GABAergic terminal. Inhibition of GABAergic synaptic neurotransmission onto dopaminergic neurons in the VTA results in activation of the mesolimbic dopaminergic circuitry, leading to a reinstatement of extinguished cocaine CPP, modelling cocaine relapse in humans. Finally, 2-AG is degraded by MAGL, which is located in GABAergic terminals. Thus, the MAGL inhibitor potentiates and prolongs orexin A-induced IPSC depression.

In this study, we found that orexin A inhibited GABAergic transmission onto dopaminergic neurons in VTA slices via a presynaptic mechanism. This effect was antagonized by OX1R and CB1R antagonists, potentiated and prolonged by a MAGL inhibitor, and prevented by an internal G protein inhibitor as well as by PLC and DAGL inhibitors. Orexin A also increased the firing activity of VTA dopaminergic neurons in a CB1R-dependent manner. A 30-min-restraint stress reinstated extinguished cocaine CPP, increased the number of activated orexin neurons in the LH, but not in the PeF/DMH, and increased orexin A and 2-AG levels in the VTA. This stress-reinstated cocaine CPP was prevented by either systemic or i.vta. injection of an OX1R or CB1R antagonist, or a DAGL inhibitor, and mimicked by i.vta. orexin A. It was also abolished in *Cnr1*-deficient mice. These results suggest that (Fig. 8) acute restraint stress activates orexin neurons in the LH, which send projections to the VTA, releasing orexins that activate postsynaptic OX1Rs on VTA dopaminergic neurons, through a PLC and DAGL enzymatic cascade to produce 2-AG. 2-AG then retrogradely inhibits GABA release through presynaptic CB1 receptors, leading to disinhibition of VTA dopaminergic neurons. 2-AG is hydrolysed by presynaptic MAGL to terminate its actions. This provides a novel mechanism for stress-induced cocaine relapse.

Orexin A-induced GABAergic IPSC depression in VTA dopaminergic neurons was antagonized by SB-334867, but not by TCS-OX2-29, suggesting that OX1Rs, but not OX2Rs, are involved. The effectiveness of AM 251 in reversing orexin A-depressed IPSCs suggests an involvement of CB1Rs, which have been demonstrated on GABAergic terminals in the VTA (ref. 30). The results that orexin A increased the PPR of paired IPSCs and decreased mIPSC frequency further support a presynaptic origin of the IPSC depression. The findings that orexin A-induced IPSC depression were prevented by intracellularly applied GDP-β-S, which inhibits G-proteins, and a PLC inhibitor, edelfosine, support the involvement of G_q_PCRs and PLC. Inhibition of DAGL and MAGL, the enzymes for 2-AG synthesis and degradation^33^, respectively, prevented and potentiated the effect of orexin A, supporting the involvement of 2-AG.

Taken together, these results suggest that orexin A indirectly decreases GABA release by engagement of presynaptic CB1Rs by 2-AG, which is synthesized from DAG by postsynaptic DAGL after PLC activation, upon acting on postsynaptic OX1Rs of VTA dopaminergic neurons (Fig. 8).

In addition to inhibiting GABA release (disinhibition) indirectly through 2-AG, orexin A could directly increase the neuronal activity of dopaminergic neurons or GABAergic interneurons in the VTA via postsynaptic depolarization^18^. However, the possibility of a net increase in GABAergic interneuron activity is unlikely since the overall effect of orexin A on VTA dopaminergic neuronal activity in brain slices is excitatory (Fig. 5). Since AM 251 significantly reversed orexin A-induced elevation of firing rate but not depolarization in dopaminergic neurons (Fig. 5), 2-AG-mediated disinhibition may be a major mechanism for the increased VTA dopaminergic activity produced by orexin A.

Our results suggest that endogenous orexins released during acute restraint stress initiate OX1R-PLC-DAGL-2-AG-CB1R-mediated disinhibition of VTA dopaminergic activity and contribute to cocaine relapse. First, restraint stress activated orexin neurons in the LH and increased orexin A and 2-AG levels in the VTA of mice. Second, the same stressor-induced reinstatement of extinguished cocaine CPP was mimicked by intra-VTA injection of orexin A in mice. Third, this stress-induced cocaine reinstatement was absent in *Cnr1*^−/−^ and *Cnr1*^+/−^ mice, which is indicative of a CB1R haploinsufficiency as shown previously^41^, and was completely prevented by inhibiting 2-AG synthesis using an intra-VTA DAGL inhibitor or by intra-VTA antagonism of OX1Rs or CB1Rs. The latter finding agrees with a study in rats^42^ that intra-VTA injection of either AM 251 or SB-334867 produced comparable and non-additive reductions in LH stimulation-induced CPP, suggesting that activating OX1Rs and CB1Rs in the VTA initiates a sequential cascade, rather than two parallel pathways. Our findings in VTA slices support that this sequential cascade is the OX1R-PLC-DAGL-2-AG-CB1R-mediated disinhibition mechanism in VTA dopaminergic neurons.

Orexin neurons have been reported to be dichotomously involved in arousal and reward-seeking^13^. Reward-seeking is primarily associated with LH orexin neurons, whereas arousal- and foot-shock stress-related processes are more associated with orexin neurons in the PeF/DMH. Our findings that the number of activated orexin neurons in the LH, but not PeF or DMH, was increased in cocaine-preferring mice, is in agreement with the findings of Aston-Jones *et al*.^40^. That the number of activated orexin neurons was reduced to the pre-conditioning level in extinguished mice supports the contribution of LH orexin neurons in cocaine seeking^6^. Importantly, we found that a 30-min restraint stress that reinstated extinguished cocaine CPP also activated the orexin neurons in the LH, but not PeF or DMH. Furthermore, the number of activated orexin neurons in this brain region was positively correlated with the degree of cocaine reinstatement.

Activation of LH orexin neurons has been reported to be associated with cue- and drug-reinstated drug seeking^13^. Here, we showed that LH orexin neurons may contribute to cocaine relapse through the VTA induced by acute restraint stress, a minor stressor with a strong psychological component^43^. Interestingly, Wang *et al*.^14^ reported that cocaine seeking reinstated by foot-shock stress in rat was orexin-independent in the VTA. This may be because foot-shock stimulation can activate orexin neurons in the PeF/DMH that project to areas other than the VTA, but does not activate the LH orexin neurons that project to the VTA (ref. 13). Nevertheless, Boutrel *et al*.^15^ found that orexins are involved in foot-shock stress-reinstated cocaine seeking in rats. This discrepancy may be explained as in the study of Boutrel *et al*.^15^, SB-334867 was given by systemic injection and orexin receptors in the brain regions other than the VTA may be involved^44^.

The VTA has been shown to be important for orexin-induced reinstatement of drug seeking, and OX1Rs, but not OX2Rs, are involved^14^. Here, we reported that orexin A levels are increased in the VTA in an animal model mimicking cocaine relapse, acute restraint stress-induced relapse. Restraint stress has been reported to activate hypothalamic orexin neurons in the LH and PeF of rats^36^, and the PeF in mice^37^. We further showed that acute restraint stress activated orexin neurons in the LH, but not PeF/DMH, of mice and increased orexin A levels in their VTA.

In conclusion, we revealed a disinhibition mechanism for VTA dopaminergic activity mediated by the OX1R-PLC-DAGL-2-AG-CB1R signalling cascade, which may contribute to restraint stress-induced cocaine relapse. It remains to be elucidated if this mechanism also contributes to drug relapse induced by cue, context or the drug.

## Methods

All experiments adhered to the guidelines approved by the Institutional Animal Care and Use Committees in College of Medicine, National Taiwan University and National Health Research Institutes, respectively.

### Electrophysiology study

Horizontal midbrain slices (250 μm) containing the VTA were dissected from 18- to 30-day-old Wistar rats as described previously^45^. A dissection solution containing (in mM) 110 choline, 25 NaHCO_3_, 25 D-glucose, 11.6 Na ascorbate, 7 MgSO_4_, 3.1 Na pyruvate, 2.5 KCl, 1.25 NaH_2_PO_4_ and 0.5 CaCl_2_ were used to keep dopaminergic neurons, which are vulnerable to oxidative stress, healthy^46^. After dissection, slices were equilibrated in an artificial cerebral spinal fluid (aCSF) at room temperature for at least 1 h before recording. The aCSF consisted of (in mM): 117 NaCl, 4.5 KCl, 2.5 CaCl_2_ , 1.2 MgCl_2_ , 1.2 NaH_2_PO_4_ , 25 NaHCO_3_, and 11.4 glucose and was oxygenated with 95% O_2_/5% CO_2_ , pH 7.4. During recordings, a slice was mounted in a submerged recording chamber and continuously perfused with oxygenated aCSF at a rate of 3–4 ml min^−1^ at room temperature.

Visualized whole-cell patch-clamp recordings were performed in dopaminergic neurons in VTA slices (Supplementary Fig. 1) under a stage-fixed upright infrared differential interference contrast (IR-DIC) microscope (BX51WI, Olympus, Tokyo, Japan) equipped with a × 40 water-immersion objective as reported in our previous study^24^. For voltage clamp recordings, the microelectrode (4–8 MΩ) was filled with the internal solution consisting of (in mM): 80 K^+^ gluconate, 50 KCl, 0.5 CaCl_2_, 5 BAPTA, 10 HEPES, 5 MgATP and 0.33 GTP-Tris, pH 7.4 with the liquid junction potential at 9.9 mV. When applicable, the internal solution containing 3% biocytin or 1 mM GDP-β-S was prepared by directly adding the compound into the internal solution without adjusting osmolarity or pH. For current clamp and cell-attached recordings, the internal solution was (in mM): 125 K^+^ gluconate, 5 KCl, 0.5 CaCl_2_, 5 BAPTA, 10 HEPES, 5 MgATP and 0.33 GTP-Tris (pH 7.4, with KOH) with the liquid junction potential at 11.4 mV.

### Hyperpolarization-activated inward (I h) current

To elicit I h currents, the neuron was held at −70 mV, and a series of 10 mV-increment hyperpolarization steps from −50 to −140 mV were applied for 1 s. Dopaminergic neurons were identified by the presence of a steady-state Ih current elicited from −70 to −140 mV that was >200 pA.

IPSCs and EPSCs were evoked at 0.05 Hz by 150 μs-wide pulses from a Grass stimulator (Grass Technologies, RI) through a bipolar concentric electrode (FHC, ME, which was placed 200–300 μm rostral to the recording electrode to locally stimulate afferent fibres^16^. IPSCs were recorded at −70 mV in the presence of 2 mM kynurenic acid, a non-selective glutamate receptor antagonist. mIPSCs were recorded in the presence of 1 μM tetrodotoxin. To measure the PPR of IPSCs, paired IPSCs were evoked by 50 ms-separated paired pulses every 20 s. The PPR in each neuron was the ratio of averaged amplitude of the 2nd IPSC to that of the first IPSC of three paired IPSCs. EPSCs were recorded at −70 mV in the presence of 10 μM bicuculline, a GABA_A_ receptor blocker.

To confirm the electrophysiological findings using rat slices are also present in VTA slices prepared from mice, as the latter species were used for restraint stress studies, IPSCs were also recorded in dopaminergic neurons in VTA slices dissected from 6–8-week-old mice (C57BL/6; Supplementary Fig. 4).

To monitor the neuronal firing activity and membrane potential, both whole-cell current clamp and cell-attached recordings were used. The firing rate of dopaminergic neurons was quantified in the whole cell and cell-attached recording mode, as prolonged recording in the whole cell mode may confound neuronal firing rate due to intracellular component dialysis^35^. Current clamp mode (*I*=0) was used for the whole cell or cell-attached recording experiments. Once whole cell or cell attached configuration was achieved, the recording was maintained for at least 10 min until a stable firing rate was obtained. Then, the mean neuronal firing rate over the next 2 min was recorded as the baseline. The membrane potential of pacemaker-like dopaminergic neurons, recorded in the whole-cell current clamp, was determined by the median value of diastolic depolarization between spikes^45^ (thin dotted lines in Fig. 5a). Data acquisition and analysis in electrophysiological studies were performed as described in our previous study^24^.

### TH immunofluorescence in VTA slices

After recording, the recorded neuron was filled with biocytin that had been added in the internal solution through the recording electrode. Then, the slice containing the biocytin-filled neuron was fixed in 4% paraformaldehyde (PFA) for 2 h, and washed five times with phosphate buffer saline (PBS, 0.1M, pH 7.4). Collected slices were blocked in PBS containing 0.3% (v/v) Triton X 100, 0.2% BSA and 5% normal goat serum for 2 h at room temperature. Next, slices were agitated for 48 h at 4 °C with rabbit anti-TH polyclonal antibody (1:100, Cat: 2129-S Lot.:YE052105, Epitomics. Burlingame, CA), and washed thoroughly in a PBS with 0.3% Triton X 100 and 0.2% (w/v) BSA before being agitated overnight at 4 °C with rhodamine conjugated anti-rabbit secondary antibody (1:100, Cat: AP182R Lot: 2036086, Millipore, Billerica, MA), and Alexa Fluor 488 labelled streptavidin (1:100, Cat: S11223, Lot: 1037281 Life Technologies, Grand Island, NY). Sections were mounted on slides using VectaShield anti-fade mounting media (Vector Laboratories, Inc Burlingame, CA) and visualized under a Zeiss AXIO imager microscope (Carl Zeiss Microscopy, Thornwood, NY). In all sections, it was required that the TH antibody-labelled neighbouring neurons were at the same depth in the slice as the biocytin-filled neuron to determine if the recorded neuron was TH-positive or not^47^.

### Cocaine conditioned place preference

Male C57BL/6 mice were used for the CPP test. *Cnr1* deficient (*Cnr1*^−/−^ and *Cnr1*^+/−^) mice generated as reported previously^48^ were bred in the animal room of National Health Research Institute, Chu-Nan, Miaoli, Taiwan. Adult WT and *Cnr1* deficient mice (8–14 weeks old) weighing 20–30 g were used. Mice were housed in groups of 10 in clear plastic cages and maintained in a holding room with 12:12 light–dark cycle with food and water *ad libitum*. Before conducting experiments, mice were moved in their home cages to a behavioural room and acclimated there for 1 h.

A biased cocaine CPP design as described in the previous study^49^ with modifications was conducted in mice to mimic four stages of cocaine seeking: pre-conditioning (PRE), cocaine conditioning (CON), extinction (EXT) and relapse (reinstatement (ReInstate), Fig. 6a). The CPP apparatus consisted of two distinct (black and white) chambers with equal size (13 × 13 × 13 cm^3^), separated by an intermediate compartment (7.2 × 7.2 × 13 cm^3^) with an opening to each chamber. The black chamber was equipped with a wide mesh (6 × 6 mm^2^) floor and the white chamber with a narrow grid (3 mm) floor.

On day 1, the PRE stage, the mouse was allowed to move freely between black and white chambers for 10 min. The time spent in either black or white chamber for each mouse was used for grouping mice with approximately equal bias for black or white chamber preference. Mice were excluded if the difference spent in either chamber was <100 s, that is, displaying specific chamber preference, or if they spent over 4 min in neutral area at the PRE stage. On day 2–4, the CON stage, the mouse was given an intra-peritoneal (i.p.) saline injection and confined in its preferred chamber for 30 min. Eight hours later, the same mouse was given cocaine hydrochloride (20 mg kg^−1^, i.p.) and confined in its non-preferred chamber for 30 min. On day 5, the mouse was tested for its preference for the two chambers. For each mouse, the CPP score, defined by the difference in time spent between cocaine-paired and saline-paired chambers, was used to access the degree of cocaine preference. Only mice with the CPP score >100 s, that is, being successfully cocaine-conditioned, were included in the study.

### Extinction of cocaine CPP

On day 6–8, daily extinction training was introduced to cocaine-conditioned mice. Animals received saline injections twice a day and were placed in either chamber randomly, and were tested for preference on day 9. Successful extinction of cocaine CPP was defined as the CPP score <100 s.

### Acute restraint stress-induced reinstatement of extinguished cocaine CPP

On day 10, one day after extinction, mice that had been extinguished were subjected to restraint stress-induced reinstatement of cocaine CPP. These extinguished mice were randomly separated into restrained and non-restrained groups. Mice in the restrained group were put into a 50-ml-centrifuge tube with several small holes, which kept the mouse from overheating, for 30 min. Mice in the non-restrained group remained in their home cages for an equivalent 30 min. Then, the CPP test was conducted. Mice were defined to be reinstated if the CPP score after restraint stress were >100 s.

Cocaine CPP was successfully induced by daily cocaine pairing for 3 days in 27 out of 30 naïve mice tested (90%). The established cocaine CPP was successfully extinguished after a forced extinction training by daily pairing with saline for 3 days in 26 out of 27 cocaine-preferred mice. After the extinction training, a 30-min restraint stress significantly reinstated the extinguished cocaine CPP in 20 out of 27 (74%) extinguished mice.

### Orexin A-induced reinstatement of extinguished cocaine CPP

In a group of mice, orexin A was given by bilateral intra-VTA microinjection (i.vta.) to reinstate extinguished cocaine CPP. Mice received the same cocaine-conditioning and extinction training procedures as in the stress group above except the 30-min restraint stress was replaced by an i.vta. microinjection of orexin A. The CPP test was performed 30 min after i.vta. orexin A.

### Bilateral intra-VTA cannulation

Mice received bilateral intra-VTA cannulation under isoflurane anaesthesia (1.5% for induction and 1% for maintenance) with a heated (40 °C) pad to prevent hypothermia. Briefly, animals were placed in a stereotaxic apparatus and held in a prone position with ear bar adaptors. A 26-gauge guide cannula was implanted into the VTA bilaterally according to stereotaxic coordinates: anterior-posterior (AP): 3.0 mm from Bregma; dorsal-ventral (DV): 4.6 mm from the skull surface; median-lateral coordinates (ML): 0.5 mm from the midline. (ref. 50) from bregma. The guide cannula was fixed to the skull with dental acrylic. A 33-gauge stylet was inserted into the guide cannula to maintain patency. Mice were allowed to recover from surgery for at least 4 days before experiments.

### Drug administration

For i.vta. microinjection, drug solutions of 0.2 μl were slowly injected into the VTA of mice under isoflurane (2%) anaethesia through a 33-gauge injection cannula via a 1-μl Hamilton syringe connected to a microinfusion pump (KDS311; KD Scientific) for 60 s with a further hold time of 240 s. After injection, mice were allowed to completely recover from anaesthesia for 10 min. Restraint stress was administered 30 min after i.vta. drug microinjection. The injection site was confirmed by injecting a trypan blue solution after the CPP test, the successful rate could up to 90% of total cannulated mice (Supplementary Fig 5). Data from mice with an injection site outside of the VTA (offsite injections), being <20% of total cannulated mice, were discarded.

For systemic administration, SB-334867 and AM 251 were dissolved in an aqueous solution containing 10% (w/v) encapsin and 2% (v/v) dimethylsulfoxide (DMSO). Drug solutions and the vehicle were given to the mice (0.01 ml g^−1^) by i.p. injection 30 min before restraint stress.

### Double immunohistochemical staining

Two hours after performing the CPP test at each stage, mice were anaesthetized and transcardially perfused with cold PBS followed by 4% paraformaldehyde in PBS. The brain was removed and postfixed in 4% PFA. Coronal hypothalamic sections (50 μm) were prepared for double immunohistochemical staining of c-fos and orexin A as reported previously^51^. Brain sections were first processed for Fos-immunohistochemical staining with rabbit anti-c-fos antibody (1: 500, Lot No. K 2513, Cat. No. SC-253, Santa Cruz Bio Technology, Santa Cruz, CA; biotinylated secondary, 1: 200) with nickel ammonium sulphate intensification of 3,3′-diaminobenzidine (DAB) (Vector laboratories), and then processed for orexin A (goat anti-orexin A antibody, 1: 200, Lot No. K G0813, Cat. No. SC-8070, Santa Cruz Bio Technology; biotinylated secondary, 1: 200) using the DAB without nickel ammonium sulphate as the chromagen. Slices were mounted on glass slides, and sealed with a coverslip. Double-labelled cells were readily identified because orexin stained the cytoplasm brown and *Fos* immunoreactive nuclei were stained black. For counting, two sections from each animal were chosen at the same levels with equivalent numbers of orexin-positive neurons. The Stereo Investigator software (Version 3.0, MicroBrightField, Williston, VT) was used to perform stereological analysis of orexin A single-labelled (OX^+^) and c-fos and orexin A double-labelled (Fos^+^OX^+^) neurons. All orexin-labelled neurons lateral to the fornix were considered to be in the LH. All orexin labelled neurons located dorsal and 0.4 mm medial to the fornix were considered to be in the PeF/DMH.

### Measurement of orexin A levels in the VTA

After one of four stages in our cocaine seeking model, mice were killed by decapitation. The brain was rapidly dissected and placed on its dorsal surface in a pre-cooled stainless steel adult mouse brain slicer matrix (Roboz Surgical Instrument, Gaithersburg, MD), and sliced into 1-mm-thick coronal sections. The matrix was kept in an ice bath throughout the slicing procedure. The brain tissues of the VTA were bilaterally punched out with a 0.5-mm-tip Harris Micro-Punch tool (Ted Pella Inc, Redding, CA), according to the mouse brain atlas of Paxinos and Franklin (2001)^52^.

Next, the brain tissue samples were homogenized in ice-cold lysis buffer, which contained 50 mM Tris-HCl, 150 mM NaCl, 1% nonylphenoxypolyethoxylethanol, 1 mM EDTA and 0.25% sodium deoxycholate with phosphatase and protease inhibitor cocktails (PhosSTOP, and Complete mini, Roche, Germany, respectively), using an ultrasonicator (VCX 750; Sonics & Materials, Newtown, CT) for 2 min. The supernatants were collected for the subsequent quantification of orexin A levels.

Orexin A levels in VTA homogenates were measured using a commercially available competitive chemiluminescent EIA kit (Cat: CEK-003-30, Lot: 604124, Phoenix Pharmaceuticals) according to the vendor's manual. Briefly, samples in the immunoplate, which had been coated with secondary antibody, were incubated with the primary antibody overnight at 4 °C, followed by incubation with the biotinylated secondary antibody for 1.5 h and streptavidin-horseradish peroxidase for 1 h at room temperature. Then, the substrate solution was added to develop luminescence. The luminescence intensity in each well was measured by a luminescence plate reader (Synergy HT Multi-Mode Microplate Reader, Bio-Tek Instruments, Winooski, VT). The orexin A level in each sample was calculated via interpolation from the standard curve established by various concentrations of standard solutions. Using this kit, orexin A can be reliably quantified in the linear range of 9.6–148 pg ml^−1^. Protein content in the VTA homogenates was measured using a bicinchoninc acid protein assay kit (Novagen, Darmstadt, Germany). The orexin A level in each VTA homogenate prepared was normalized to protein content.

### Measurement of endocannabinoid levels in the VTA

Lipid extractions and partial purification of the VTA tissue were performed as previously described for multiple brain areas^53^,^54^. Frozen tissue was subjected to a 40:1 methanolic extraction that was spiked with 100 pmol of deuterium-labelled *N*-arachidonoyl glycine as an internal standard. Parafilm-covered tubes with this preparation were maintained on ice and shielded from light for ∼2 h. While in an ice-slurry, samples were homogenized using a polytron for ∼1 min. Samples were centrifuged at 19,000*g* at 24 °C for 20 min. Supernatants were collected and placed in polypropylene tubes (15 ml) and high-performance liquid chromatography (HPLC)-grade water was added to make a final supernatant/water solution 25% organic. This mixture was subjected to partial purification on a Preppy apparatus (Sigma-Aldrich) assembled with 500 mg C18 solid-phase extraction columns (Agilent Technologies, Santa Clara, CA). Columns were conditioned with 5 ml of HPLC-grade methanol immediately followed by 2.5 ml of HPLC-grade water. The supernatant/water solution was then loaded onto the C18 SPE column then washed with 2.5 ml of HPLC grade water followed by 1.5 ml of 40% methanol. Elutions from 60, 70, 85 and 100% methanol were collected in individual autosampler vials and then stored in a −80 °C freezer until mass spectrometer analysis.

Samples were removed from the −80 °C freezer and allowed to warm to room temperature (∼15 min), vortexed for ∼1 min, then put into the autosampler, which is maintained at 24 °C (Agilent 1100 series autosampler, Palo Alto, CA) for LC/MS/MS analysis. Rapid separation of eluants of 20 μl occurred using a C18 Zorbax reversed-phase analytical column (Agilent Technologies, Santa Clara, CA) to scan for individual compounds (mobile phase A: 20% HPLC methanol, 80% HPLC water, 1 mM ammonium acetate; mobile phase B: 100% HPLC methanol, 1 mM ammonium acetate). Gradient elution (200 μl min^−1^) then occurred under the pressure created by two Shimadzu 10AdVP pumps (Columbia, MD). Next, electrospray ionization was accomplished using an Applied Biosystems/MDS Sciex (Foster City, CA) API3000 triple quadrupole mass spectrometer. Multiple reaction monitoring coupled to LC/MS/MS was used to analyse levels of each compound present in the sample injection. Synthetic standards were used to generate optimized multiple reaction monitoring methods and standard curves were generated for analysis of total content of each unknown amount as previously described^53^.

### Drugs

Orexin A, SB-334867 (1-(2-methylbenzoxazol-6-yl)-3-[1,5]naphthyridin-4-yl urea), TCS-OX2-29 ((2S)-1-(3,4-Dihydro-6,7-dimethoxy-2(1H)-isoquinolinyl)-3,3-dimethyl -2-[(4-pyridinylmethyl)amino]-1-butanone hydrochloride), Edelfosine ((7R)-4-Hydroxy -7- methoxy-N,N,N-trimethyl-3,5,9-trioxa-4-phosphaheptacosan-1-aminium-4-oxide) and JZL 184 (4-[Bis(1,3-benzodioxol-5-yl)hydroxymethyl]-1-piperidinecarboxylic acid 4-nitrophenyl ester) were purchased from Tocris Bioscience (Bristol, U.K). AM 251 (1-(2,4-Dichlorophenyl)-5-(4-iodophenyl)-4-methyl-N-(1-piperidyl)pyrazole-3-carboxaide), (−)-THL, WIN 55212-2 ((R)-(+)-[2,3-Dihydro-5-methyl-3 [(4-morpholinyl)methyl]pyrrolo[1,2,3-de]-1,4-benzoxazinyl]-(1-naphthalenyl) methanone mesylate salt), GDP-β-S (Guanosine 5′-[β-thio]diphosphate trilithium salt) and biocytin were purchased from Sigma-Aldrich (St Louis, MO). Kynurenic acid was purchased from Ascent Scientific (North Somerset, U.K). Cocaine hydrochloride was purchased under the approval from Food and Drug Administration, the Ministry of Health and Welfare, Taiwan. For electrophysiological studies, all drugs were prepared as a 1,000-fold concentrated stock solution and diluted to their final concentration with aCSF. Orexin A and TCS-OX2-29 we dissolved in deionized water. Kynurenic acid was dissolved in aCSF directly before use. SB-334867, THL, WIN 55212-2, JZL184 and AM 251 were dissolved in DMSO. Edelfosine was dissolved in anhydrous ethanol. The final concentration of DMSO was <0.1% and the final ethanol concentration was 0.04%, which had no effect *per se*. For *in vivo* i.p. injections, SB 334867 and AM251 were dissolved in a water solution containing 10% (w/v) encapsin and 2% (v/v) DMSO. For i.vta. microinjections, orexin A was dissolved in saline and SB-334867, AM 251 and THL were dissolve in DMSO. Cocaine was dissolved in saline. All drugs were prepared as the working concentrations for either i.p. or i.vta. injection.

### Data analysis

The *n* numbers refer to the number of recorded neurons or the number of animals. Before conducting the experiments, the sample size for each group was estimated with an expected power of 90% using the s.d. values from a pilot study in rat or mice. Statistical comparisons for *in vitro* studies between different treated groups were analysed by Student's *t*-tests. Mann–Whitney *U* test used for analysing the nonparametric data of 2-AG level, for those data within the same neuron with different treatments by paired *t* test, one-sample *t* test for normalized data. Repeated measures one-way ANOVA with Dunns *post hoc* test was used for analysing the nonparametric data of neuronal firing rate. For the electrophysiological studies, 3–4 slices were dissected from one animal. Usually, one neuron was recorded from 1 slice, and 1–2 slices were sampled from one animal. For behavioural data, in our cocaine-CPP model, most of data in each stage are normally distributed. Animals were randomly allocated into different groups. All the measurements are objective data. The experimenters were not blinded to the group allocation of the mice. Therefore, differences among different treatment groups were analysed by two-way ANOVA with repeat measures over stage. Differences between two groups at each stage were analysed by Bonferroni's *post hoc* test. *P* values <0.05 (two-sided test) were considered statistically significant.

Most of the data of c-fos-expressing LH orexin neuronal numbers and VTA orexin A levels were normally distributed with equal variance and an expected power of 90%. Therefore, one-way ANOVA followed by Newman–Keuls *post hoc* test was used for these experimental data. *P* values<0.05 were considered statistically significant. Pearson's *r* correlation was used to analyse the association between the degree of cocaine relapse and the number of activated LH orexin neurons.

### Data availability

The authors declare that the data supporting the findings of this study are included within the article and its Supplementary information files, or are available from the authors on request.

## Additional information

**How to cite this article:** Tung, L.W. *et al*. Orexins contribute to restraint stress-induced cocaine relapse by endocannabinoid-mediated disinhibition of dopaminergic neurons. *Nat. Commun.* 7:12199 doi: 10.1038/ncomms12199 (2016).

## Supplementary Material

Supplementary Information Supplementary Figures 1-5, Supplementary Table 1

## References

[b1] Epstein D. H., Preston K. L., Stewart J. & Shaham Y. Toward a model of drug relapse: an assessment of the validity of the reinstatement procedure. Psychopharmacology (Berl) 189, 1–16 (2006). 17019567 10.1007/s00213-006-0529-6 PMC1618790

[b2] Katz J. L. & Higgins S. T. The validity of the reinstatement model of craving and relapse to drug use. Psychopharmacology (Berl) 168, 21–30 (2003). 12695875 10.1007/s00213-003-1441-y

[b3] Sakurai T. . Orexins and orexin receptors: a family of hypothalamic neuropeptides and G protein-coupled receptors that regulate feeding behavior. Cell 92, 573–585 (1998). 9491897 10.1016/s0092-8674(00)80949-6

[b4] de Lecea L. . The hypocretins: hypothalamus-specific peptides with neuroexcitatory activity. Proc. Natl Acad. Sci. USA 95, 322–327 (1998). 9419374 10.1073/pnas.95.1.322 PMC18213

[b5] Wong K. K., Ng S. Y., Lee L. T., Ng H. K. & Chow B. K. Orexins and their receptors from fish to mammals: a comparative approach. Gen. Comp. Endocrinol. 171, 124–130 (2011). 21216246 10.1016/j.ygcen.2011.01.001

[b6] Mahler S. V., Smith R. J., Moorman D. E., Sartor G. C. & Aston-Jones G. Multiple roles for orexin/hypocretin in addiction. Prog. Brain Res. 198, 79–121 (2012). 22813971 10.1016/B978-0-444-59489-1.00007-0 PMC3643893

[b7] Chiou L. C. . Orexins/hypocretins: pain regulation and cellular actions. Curr. Pharm. Des. 16, 3089–3100 (2010). 20687883 10.2174/138161210793292483

[b8] Kukkonen J. P. Physiology of the orexinergic/hypocretinergic system: a revisit in 2012. Am. J. Physiol. Cell Physiol. 304, C2–32 (2012). 23034387 10.1152/ajpcell.00227.2012

[b9] Harris G. C., Wimmer M. & Aston-Jones G. A role for lateral hypothalamic orexin neurons in reward seeking. Nature 437, 556–559 (2005). 16100511 10.1038/nature04071

[b10] Baimel C. . Orexin/hypocretin role in reward: implications for opioid and other addictions. Br. J. Pharmacol. 172, 334–348 (2014). 24641197 10.1111/bph.12639 PMC4292951

[b11] Fadel J. & Deutch A. Y. Anatomical substrates of orexin-dopamine interactions: lateral hypothalamic projections to the ventral tegmental area. Neuroscience 111, 379–387 (2002). 11983323 10.1016/s0306-4522(02)00017-9

[b12] Wise R. A. Addictive drugs and brain stimulation reward. Annu. Rev. Neurosci. 19, 319–340 (1996). 8833446 10.1146/annurev.ne.19.030196.001535

[b13] Harris G. C. & Aston-Jones G. Arousal and reward: a dichotomy in orexin function. Trends Neurosci. 29, 571–577 (2006). 16904760 10.1016/j.tins.2006.08.002

[b14] Wang B., You Z. B. & Wise R. A. Reinstatement of cocaine seeking by hypocretin (orexin) in the ventral tegmental area: independence from the local corticotropin-releasing factor network. Biol. Psychiatry 65, 857–862 (2009). 19251246 10.1016/j.biopsych.2009.01.018 PMC2705875

[b15] Boutrel B. . Role for hypocretin in mediating stress-induced reinstatement of cocaine-seeking behavior. Proc. Natl Acad. Sci. USA 102, 19168–19173 (2005). 16357203 10.1073/pnas.0507480102 PMC1323172

[b16] Borgland S. L., Taha S. A., Sarti F., Fields H. L. & Bonci A. Orexin A in the VTA is critical for the induction of synaptic plasticity and behavioral sensitization to cocaine. Neuron 49, 589–601 (2006). 16476667 10.1016/j.neuron.2006.01.016

[b17] Aston-Jones G. . Lateral hypothalamic orexin/hypocretin neurons: A role in reward-seeking and addiction. Brain Res. 1314, 74–90 (2010). 19815001 10.1016/j.brainres.2009.09.106 PMC2819557

[b18] Korotkova T. M., Sergeeva O. A., Eriksson K. S., Haas H. L. & Brown R. E. Excitation of ventral tegmental area dopaminergic and nondopaminergic neurons by orexins/hypocretins. J. Neurosci. 23, 7–11 (2003). 12514194 10.1523/JNEUROSCI.23-01-00007.2003 PMC6742159

[b19] Baimel C. & Borgland S. L. Orexin signaling in the VTA gates morphine-induced synaptic plasticity. J. Neurosci. 35, 7295–7303 (2015). 25948277 10.1523/JNEUROSCI.4385-14.2015 PMC6605267

[b20] De Vries T. J. . A cannabinoid mechanism in relapse to cocaine seeking. Nat. Med. 7, 1151–1154 (2001). 11590440 10.1038/nm1001-1151

[b21] Fattore L., Spano M. S., Cossu G., Deiana S. & Fratta W. Cannabinoid mechanism in reinstatement of heroin-seeking after a long period of abstinence in rats. Eur. J. Neurosci. 17, 1723–1726 (2003). 12752390 10.1046/j.1460-9568.2003.02607.x

[b22] Cippitelli A. . Cannabinoid CB1 receptor antagonism reduces conditioned reinstatement of ethanol-seeking behavior in rats. Eur. J. Neurosci. 21, 2243–2251 (2005). 15869521 10.1111/j.1460-9568.2005.04056.x

[b23] De Vries T. J., de Vries W., Janssen M. C. & Schoffelmeer A. N. Suppression of conditioned nicotine and sucrose seeking by the cannabinoid-1 receptor antagonist SR141716A. Behav. Brain Res. 161, 164–168 (2005). 15904723 10.1016/j.bbr.2005.02.021

[b24] Ho Y. C. . Activation of orexin 1 receptors in the periaqueductal gray of male rats leads to antinociception via retrograde endocannabinoid (2-arachidonoylglycerol)-induced disinhibition. J. Neurosci. 31, 14600–14610 (2011). 21994376 10.1523/JNEUROSCI.2671-11.2011 PMC3265563

[b25] Kano M., Ohno-Shosaku T., Hashimotodani Y., Uchigashima M. & Watanabe M. Endocannabinoid-mediated control of synaptic transmission. Physiol. Rev. 89, 309–380 (2009). 19126760 10.1152/physrev.00019.2008

[b26] Matyas F. . Identification of the sites of 2-arachidonoylglycerol synthesis and action imply retrograde endocannabinoid signaling at both GABAergic and glutamatergic synapses in the ventral tegmental area. Neuropharmacology 54, 95–107 (2008). 17655884 10.1016/j.neuropharm.2007.05.028 PMC2238033

[b27] Pan B., Hillard C. J. & Liu Q. S. Endocannabinoid signaling mediates cocaine-induced inhibitory synaptic plasticity in midbrain dopamine neurons. J. Neurosci. 28, 1385–1397 (2008). 18256258 10.1523/JNEUROSCI.4033-07.2008 PMC6671588

[b28] Johnson S. W. & North R. A. Two types of neurone in the rat ventral tegmental area and their synaptic inputs. J. Physiol. 450, 455–468 (1992). 1331427 10.1113/jphysiol.1992.sp019136 PMC1176131

[b29] Zucker R. S. & Regehr W. G. Short-term synaptic plasticity. Annu. Rev. Physiol. 64, 355–405 (2002). 11826273 10.1146/annurev.physiol.64.092501.114547

[b30] Riegel A. C. & Lupica C. R. Independent presynaptic and postsynaptic mechanisms regulate endocannabinoid signaling at multiple synapses in the ventral tegmental area. J. Neurosci. 24, 11070–11078 (2004). 15590923 10.1523/JNEUROSCI.3695-04.2004 PMC4857882

[b31] Zhao L. & Levine E. S. BDNF-endocannabinoid interactions at neocortical inhibitory synapses require phospholipase C signaling. J. Neurophysiol. 111, 1008–1015 (2014). 24335212 10.1152/jn.00554.2013 PMC3949235

[b32] Hadvary P., Sidler W., Meister W., Vetter W. & Wolfer H. The lipase inhibitor tetrahydrolipstatin binds covalently to the putative active site serine of pancreatic lipase. J. Biol. Chem. 266, 2021–2027 (1991). 1899234

[b33] Ludanyi A. . Complementary synaptic distribution of enzymes responsible for synthesis and inactivation of the endocannabinoid 2-arachidonoylglycerol in the human hippocampus. Neuroscience 174, 50–63 (2011). 21035522 10.1016/j.neuroscience.2010.10.062 PMC3678284

[b34] Blankman J. L., Simon G. M. & Cravatt B. F. A comprehensive profile of brain enzymes that hydrolyze the endocannabinoid 2-arachidonoylglycerol. Chem. Biol. 14, 1347–1356 (2007). 18096503 10.1016/j.chembiol.2007.11.006 PMC2692834

[b35] Alcami P., Franconville R., Llano I. & Marty A. Measuring the firing rate of high-resistance neurons with cell-attached recording. J. Neurosci. 32, 3118–3130 (2012). 22378885 10.1523/JNEUROSCI.5371-11.2012 PMC6622012

[b36] Gerashchenko D., Horvath T. L. & Xie X. S. Direct inhibition of hypocretin/orexin neurons in the lateral hypothalamus by nociceptin/orphanin FQ blocks stress-induced analgesia in rats. Neuropharmacology 60, 543–549 (2011). 21195099 10.1016/j.neuropharm.2010.12.026 PMC3031765

[b37] Winsky-Sommerer R. . Interaction between the corticotropin-releasing factor system and hypocretins (orexins): a novel circuit mediating stress response. J. Neurosci. 24, 11439–11448 (2004). 15601950 10.1523/JNEUROSCI.3459-04.2004 PMC6730356

[b38] Martin M., Ledent C., Parmentier M., Maldonado R. & Valverde O. Cocaine, but not morphine, induces conditioned place preference and sensitization to locomotor responses in CB1 knockout mice. Eur. J. Neurosci. 12, 4038–4046 (2000). 11069600 10.1046/j.1460-9568.2000.00287.x

[b39] Houchi H. . CB1 receptor knockout mice display reduced ethanol-induced conditioned place preference and increased striatal dopamine D2 receptors. Neuropsychopharmacology 30, 339–349 (2005). 15383833 10.1038/sj.npp.1300568

[b40] Harris G. C., Wimmer M., Randall-Thompson J. F. & Aston-Jones G. Lateral hypothalamic orexin neurons are critically involved in learning to associate an environment with morphine reward. Behav. Brain Res. 183, 43–51 (2007). 17599478 10.1016/j.bbr.2007.05.025 PMC2030620

[b41] Crombag H. S., Johnson A. W., Zimmer A. M., Zimmer A. & Holland P. C. Deficits in sensory-specific devaluation task performance following genetic deletions of cannabinoid (CB1) receptor. Learn. Mem. 17, 18–22 (2010). 20035014 10.1101/lm.1610510

[b42] Taslimi Z., Haghparast A., Hassanpour-Ezatti M. & Safari M. S. Chemical stimulation of the lateral hypothalamus induces conditioned place preference in rats: involvement of OX1 and CB1 receptors in the ventral tegmental area. Behav. Brain Res. 217, 41–46 (2011). 20937330 10.1016/j.bbr.2010.10.007

[b43] Nitish Bhatia . Animal models in the study of stress: A review. NSHM J. Pharm. Healthc. Manag. 02, 42–50 (2011).

[b44] Plaza-Zabala A., Maldonado R. & Berrendero F. The hypocretin/orexin system: implications for drug reward and relapse. Mol. Neurobiol. 45, 424–439 (2012). 22430644 10.1007/s12035-012-8255-z

[b45] Marinelli S., Pascucci T., Bernardi G., Puglisi-Allegra S. & Mercuri N. B. Activation of TRPV1 in the VTA excites dopaminergic neurons and increases chemical- and noxious-induced dopamine release in the nucleus accumbens. Neuropsychopharmacology 30, 864–870 (2005). 15562294 10.1038/sj.npp.1300615

[b46] Ho S. Y., Chen C. H., Liu T. H., Chang H. F. & Liou J. C. Protein kinase mzeta is necessary for cocaine-induced synaptic potentiation in the ventral tegmental area. Biol. Psychiatry 71, 706–713 (2012). 22153887 10.1016/j.biopsych.2011.10.031

[b47] Margolis E. B., Coker A. R., Driscoll J. R., Lemaitre A. I. & Fields H. L. Reliability in the identification of midbrain dopamine neurons. PLoS One 5, e15222 (2010). 21151605 10.1371/journal.pone.0015222 PMC3000317

[b48] Zimmer A., Zimmer A. M., Hohmann A. G., Herkenham M. & Bonner T. I. Increased mortality, hypoactivity, and hypoalgesia in cannabinoid CB1 receptor knockout mice. Proc. Natl Acad. Sci. USA 96, 5780–5785 (1999). 10318961 10.1073/pnas.96.10.5780 PMC21937

[b49] Tzeng W. Y. . Companions reverse stressor-induced decreases in neurogenesis and cocaine conditioning possibly by restoring BDNF and NGF levels in dentate gyrus. Psychoneuroendocrinology 38, 425–437 (2013). 22832183 10.1016/j.psyneuen.2012.07.002

[b50] Labouebe G. . Insulin induces long-term depression of ventral tegmental area dopamine neurons via endocannabinoids. Nat. Neurosci. 16, 300–308 (2013). 23354329 10.1038/nn.3321 PMC4072656

[b51] Lee H. J. . Stress induces analgesia via orexin 1 receptor-initiated endocannabinoid/CB1 signaling in the mouse periaqueductal gray. Neuropharmacology 105, 577–586 (2016). 26907809 10.1016/j.neuropharm.2016.02.018 PMC8081448

[b52] Paxinos G. & Franklin K. The Mouse Brain in Stereotaxic Coordinates (Deluxe Edition) 2nd edn Academic Press (2001).

[b53] Leishman E. . Broad impact of deleting endogenous cannabinoid hydrolyzing enzymes and the CB1 cannabinoid receptor on the endogenous cannabinoid-related lipidome in eight regions of the mouse brain. Pharmacol. Res. 110, 159–172 (2016). 27109320 10.1016/j.phrs.2016.04.020 PMC4914450

[b54] Stuart J. M., Paris J. J., Frye C. & Bradshaw H. B. Brain levels of prostaglandins, endocannabinoids, and related lipids are affected by mating strategies. Int. J. Endocrinol. 2013, 436252 (2013). 24369463 10.1155/2013/436252 PMC3863470

