# Supplementary material for: Orexins contribute to restraint stress-induced cocaine relapse by endocannabinoid-mediated disinhibition of dopaminergic neurons
Source: Nat Commun. 2016 Jul 22;7:12199. doi: 10.1038/ncomms12199 (PMC4961842; doi:10.1038/ncomms12199)
Supplement: Supplementary Information — Supplementary Figures 1-5, Supplementary Table 1 [file ncomms12199-s1.pdf]

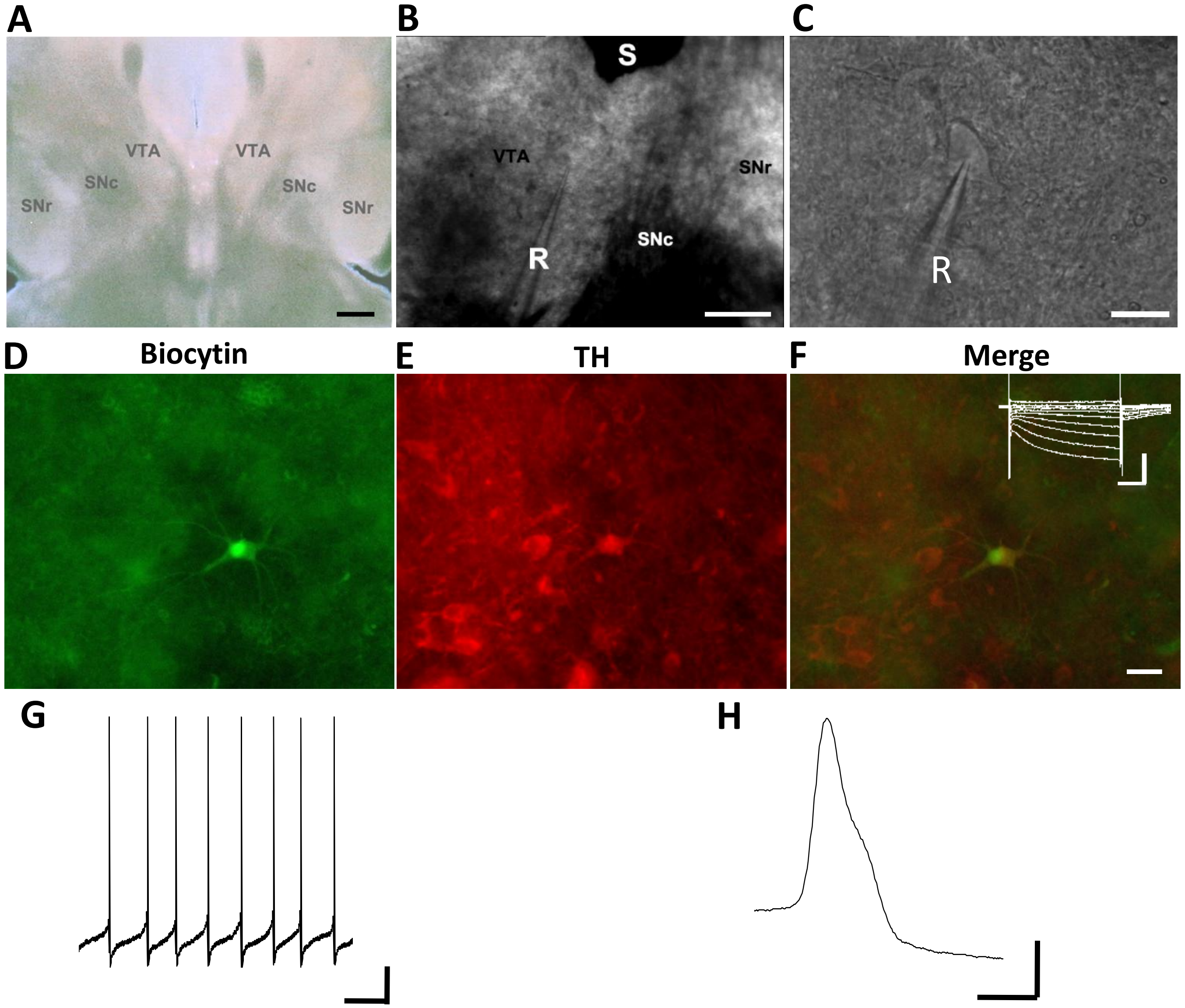

Supplemental Figure 1. Identification of dopaminergic neurons in VTA slices. (a) Micrographs demonstrate the location of the VTA with the landmark of the substantia nigra compacta (SNc) in a brain slice. Scale bar, 500 µm , **(b)** where the stimulating electrode (**S**) was placed rostral to the recording microelectrode (**R**). Scale bar, 200 µm, **(c)** when recording from a VTA neuron. Scale bar, 25 µm. **(d)** Micrographs of a recorded VTA neuron filled with biocytin and **(e)** expressing tyrosine hydroxylase (TH), and **(f)** a superimposed image. Scale bar, 40 µm. Inset in **(f)**: Membrane currents elicited by voltage steps from -70 mV to -50 to -140 mV, in -10 mV-increments in this dopaminergic neuron. Scale bars, 200 ms and 400 pA. Besides, **(g)** dopaminergic neurons have lower basal firing rates. Scale bars, 1s and 20 mV, **(h)** wider action potentials, Scale bars, 5 ms and 20 mV. and stronger firing adaptation than GABA neurons. Note that robust Ih currents were elicited by hyperpolarizing voltage steps. VTA: Ventral Tegmental Area, SNc: Substantia Nigra compacta, SNr: Substantia Nigra reticulate.

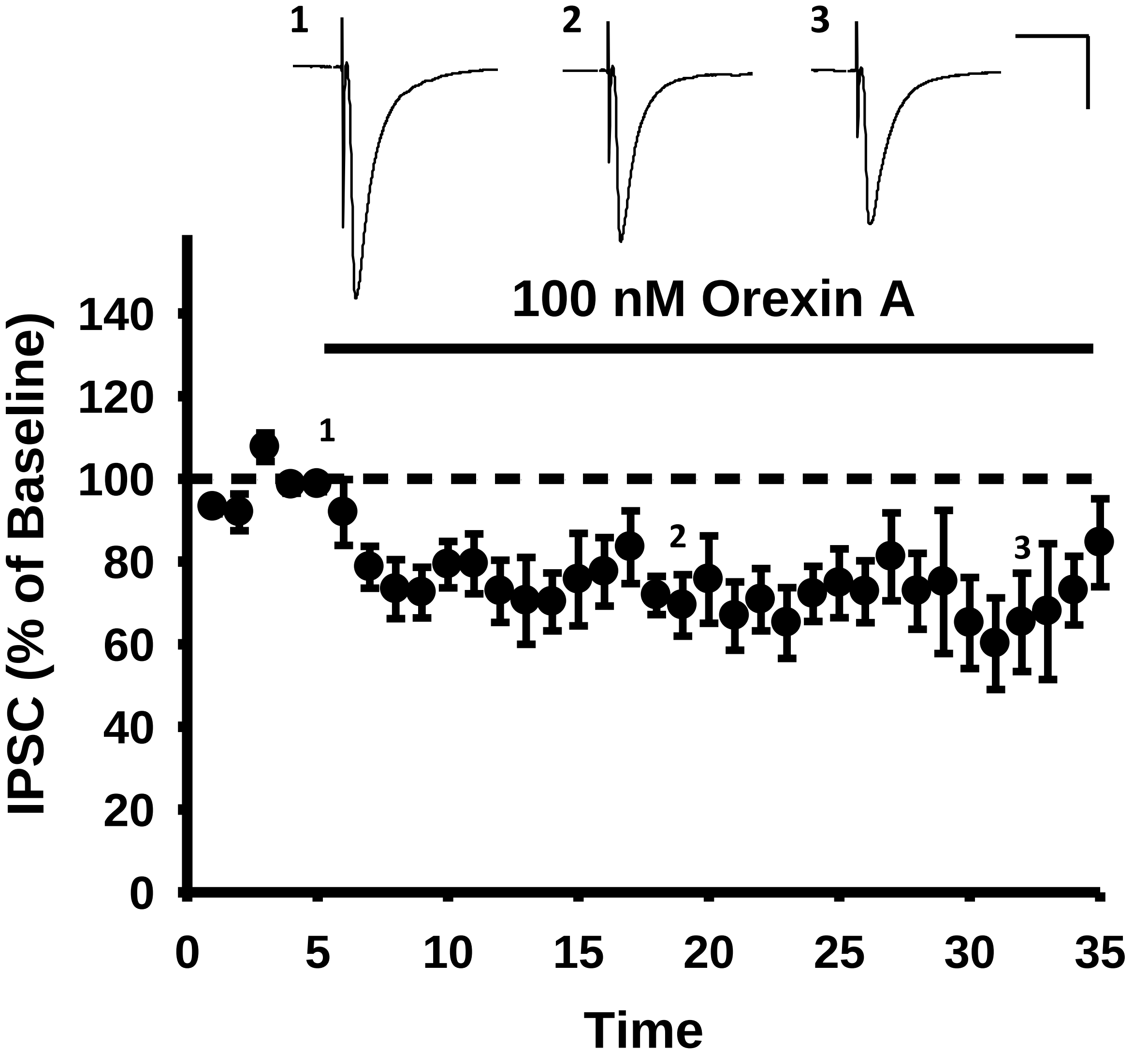

Supplemental Figure 2. Long term treatment with orexin A did not cause receptor desensitization. Time courses of effects of orexin A (100 nM) on IPSCs (n=4). Upper panels are the representative IPSC traces taken at time point 1, 2 and 3, respectively, from a neuron. Scale bars, 50 ms and 200 pA.

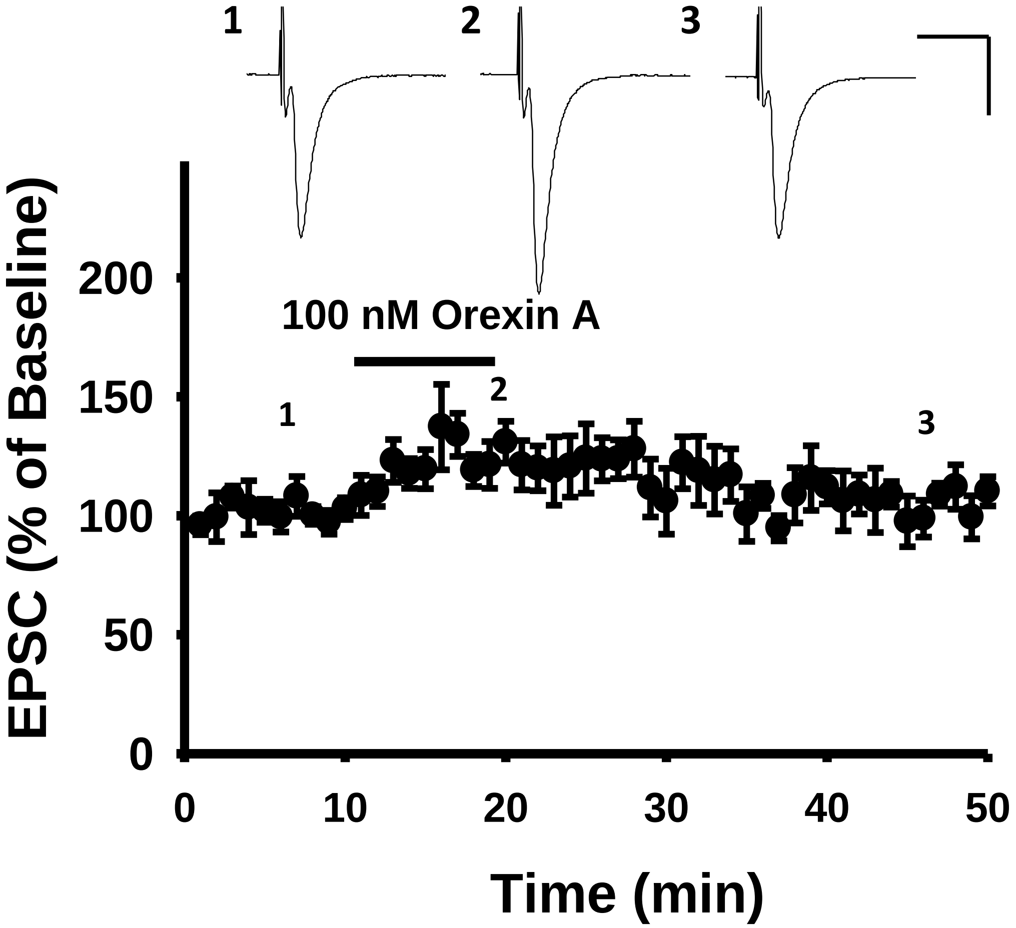

Supplemental Figure 3. Orexin A did not depress evoked EPSCs Time courses of effects of orexin A (100 nM) on EPSCs (n=5). Upper panels are the representative IPSC traces taken at time point 1, 2 and 3, respectively, from a neuron. Scale bars, 50 ms and 200 pA.

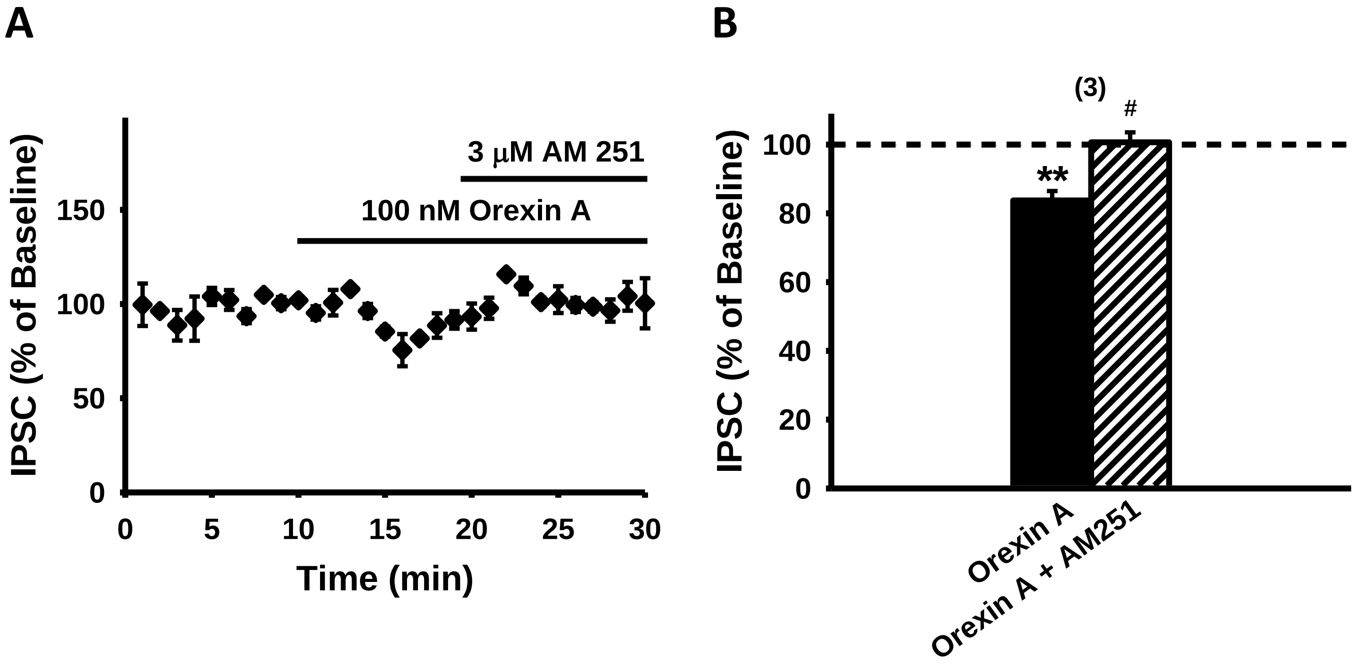

Supplemental Figure 4. Orexin A depressed IPSCs in dopaminergic neurons of mouse VTA slices in a manner reversed by a CB1R antagonist. (a) The time course of the effect of orexin A (100 nM) on the IPSC amplitude before and after treatment with AM 251 (3 μM). **(b)** The effect of orexin A (n=3) (black bar) alone or in combination with AM 251 (right slashed bar) on the IPSC amplitude. Data analysis and presentation are the same as Fig. 1. ** $p < 0.01$ (one sample *t* test); # $p < 0.05$ *vs*. orexin A alone (paired *t* test)

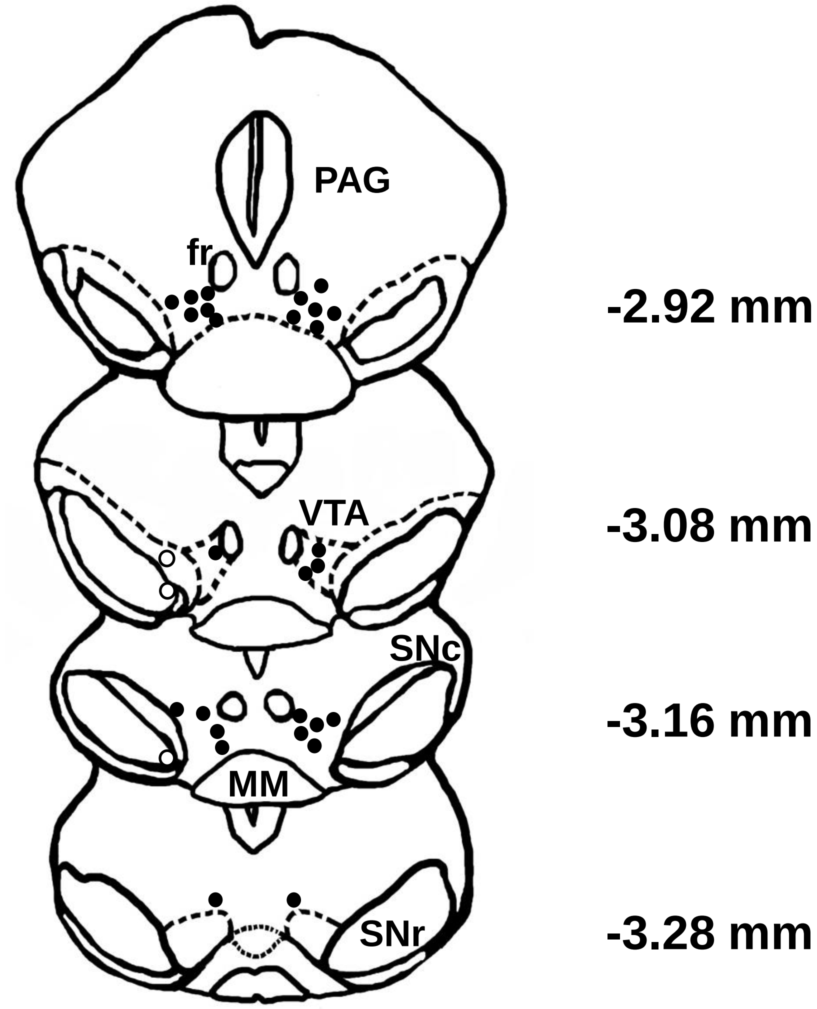

Supplemental Figure 5. Reconstructed bilateral injection sites in the VTA are shown in drawn midbrain coronal sections. Distance from bregma is shown to the right of each section (in millimeters)[1]. Injection sites from mice used in the stress induced reinstatement experiment, on site (closed circles); off site (open circles). fr: fasciculus retroflexus, MM: Medial Mammill, PAG: Periaqueductal gray, SNc: Substantia Nigra compacta, SNr: Substantia Nigra reticulate,VTA: Ventral Tegmental Area.

Supplementary Table 1. Endocannabinoid levels in the VTA of extinguished mice with and without restraint stress.

Endocannabinoids	No Stress	Stress
	(pmol/g tissue)	
2-AG	422.95 ± 59.93	547.65 ± 42.73*
2-OG	1433.21 ± 371.87	2211.19 ± 320.80
P-Gly	38.15 ± 3.78	44.13 ± 4.934
PEA	197.99 ± 43.78	159.28 ± 19.44
SEA	169.09 ± 22.16	177.09 ± 21.72
OEA	124.85 ± 33.03	86.53 ± 10.11
LEA	46.39 ± 14.15	37.83 ± 3.79

Mice were randomly divided into restrained and non-restrained groups. Both groups received the same cocaine-conditioning and extinction training in the CPP test. After extinction, the restrained, but not the non-restrained, group received a 30-min restraint stress. Brain tissue containing the VTA was quickly isolated after the restraint stress. Endocannabinoids were extracted and measured by LC/MS/MS as described in Methods. Data are presented as means ± SE. *$p < 0.05$, n=8 for each group.

2-AG, 2-arachidonyl glycerol; 2-OG, 2-oleoylglycerol; P-Gly, N-palmitoleoyl glycine; PEA, palmitoylethanolamine; SEA, stearoylethanolamine; OEA, oleoylethanolamine

and LEA, dihomo-γ-linolenoylethanolamine.

Supplementary References

1. Paxinos G, Franklin K. *The Mouse Brain in Stereotaxic Coordinates (Deluxe Edition)*, Second edn. Academic Press, California, USA (2001).
